# Protocol for Head StART: A hybrid type II cluster randomized controlled trial evaluating community ART delivery for people newly diagnosed with HIV in refugee settlements in Uganda

**DOI:** 10.1371/journal.pone.0340916

**Published:** 2026-02-27

**Authors:** Robin E. Klabbers, Ambrose Mugyenyi, Rogers Nsubuga, Gloria Asaba, Zikama Faustin, Agnes Kiragga, Laura Drown, Andrew Mujugira, Melissa Mugambi, Monisha Sharma, Paul Drain, Nicholas A. Middleton, Elinor M. Sveum, Samuel Lewis, Timothy R. Muwonge, Kelli N. O’Laughlin

**Affiliations:** 1 Department of Global Health, University of Washington, Seattle, Washington, United States of America; 2 Department of Emergency Medicine, University of Washington, Seattle, Washington, United States of America; 3 Infectious Diseases Institute, Makerere University, Kampala, Uganda; 4 African Population and Health Research Center, Nairobi, Kenya; 5 Department of Medicine, University of Washington, Seattle, Washington, United States of America; 6 Department of Epidemiology, University of Washington, Seattle, Washington, United States of America; 7 School of Medicine, University of Washington, Seattle, Washington, United States of America; PLOS: Public Library of Science, UNITED STATES OF AMERICA

## Abstract

**Background:**

Until recently, community-based antiretroviral therapy (ART) delivery was only available for people living with HIV on ART for ≥6 months with a suppressed viral load. However, early attrition from HIV care is common, particularly in refugee settlements where structural and psychosocial barriers to care are pronounced, and reducing barriers to life-saving ART sooner (e.g., at ART initiation) through community-based ART delivery may improve treatment outcomes. The Head StART study will evaluate the effectiveness, implementation, and health system costs of community ART delivery for individuals newly diagnosed with HIV in refugee settlements in Uganda.

**Methods:**

In a cluster randomized trial, twelve health centers in five refugee settlements in Uganda were randomized (1:1) to intervention or standard of care (SoC), with stratification to balance site characteristics. Individuals testing positive for HIV are screened for study eligibility (inclusion criteria: diagnosed with HIV < 6 months, on ART ≤ 42 days, ≥ 18 years of age or emancipated minor or mature minor; exclusion criteria: pregnant/breastfeeding, medical reason for requiring facility-based care, concurrent trial participation) aiming to recruit up to 1,560 participants per arm (N = 3,120 total). Eligible individuals are enrolled and monitored longitudinally through abstraction of routinely collected data on HIV care outcomes. At intervention sites, participants are offered community ART delivery (Head StART intervention); at SoC sites, participants receive facility-based care. The primary outcome compares the proportion of participants virally suppressed 12 months post-ART initiation by arm in an intention-to-treat analysis. Qualitative interviews and direct observations will augment quantitative data to evaluate intervention implementation across sites. Micro-costing, time and motion observations, and costing interviews will assess intervention costs, which will inform a budget impact analysis.

**Discussion:**

This trial will generate critical effectiveness, implementation, and budget impact data on community ART delivery for individuals newly diagnosed with HIV in refugee settlements needed to optimize HIV care for humanitarian populations.

**Trial registration:**

ClinicalTrials.gov: NCT06126913. Registered 27 September 2023. https://clinicaltrials.gov/study/NCT06126913

## Introduction

Despite the availability of free clinical HIV services including life-saving antiretroviral therapy (ART) [1] in refugee settlements in sub-Saharan Africa, engagement in care among individuals living with HIV in these humanitarian settings is low [2,3]. Humanitarian populations living in refugee settlements face unique hardships that hinder care access, including unmet basic needs, disrupted social structures, limited livelihood opportunities, threats to their security, and increased susceptibility to mental health problems [3–8].

Community ART delivery is a differentiated service delivery model for HIV designed to address barriers to HIV care engagement. In community ART delivery, HIV services are brought closer to people living with HIV (PLHIV) by distributing ART in the community instead of at the health facility. This client-centered approach lowers the time and transportation burden associated with accessing care and fosters social support through its group-based structure [9]. Although community ART delivery has not yet been studied in refugee settlements, it has demonstrated effectiveness in other contexts. Strong evidence of the benefits of community ART delivery exists, including retention in care [10–15], ART adherence [12,13,16,17], viral suppression [14,18], and peer support [12,19].

Generally, in sub-Saharan Africa, community-based ART delivery has only been available for clients who are adherent to ART for >6 months. Similarly, in Uganda, until late 2023, community ART delivery was reserved for “stable clients”, i.e., PLHIV who had spent 12 months on a 1^st^ or 2^nd^ line ART regimen, had a self-reported adherence >95%, a suppressed viral load (<1,000 copies/mL), and were classified as World Health Organization (WHO) stage 1 or 2 [20]. Individuals newly diagnosed with HIV were excluded from community ART delivery participation.

Community ART delivery, however, may be especially beneficial following a new HIV diagnosis when attrition from care is high [21–23]. In refugee settlements, individuals newly diagnosed with HIV often do not disclose their status to others, fearing stigma and social repercussions [24], and, consequently, are forced to navigate the challenges of accessing care alone. As members of community ART delivery groups, these newly diagnosed individuals would gain access to the support of other PLHIV navigating the same challenges. By fostering social support – a factor previously associated with linkage to care in refugee settlements [25] – and lowering barriers to care, community ART delivery holds potential to improve clinical outcomes and reduce secondary transmission during a time when viral load is high.

Uganda has > 1.8 million refugees and is the fifth largest refugee-hosting country in the world [26,27]. Since 2018, two forms of community ART delivery have been offered throughout Uganda, including in refugee settlements to stable clients: community client-led ART delivery (CCLAD) and community drug distribution point (CDDP). Per the Uganda Ministry of Health (MoH) guidelines, in CCLAD, PLHIV are placed into groups of 3–6 individuals, in which members rotate the responsibility for representing the group and collecting ART from the health center [20]. After receiving ART from the health center, the group representative distributes the collected ART to other group members at an agreed-upon community location. In CDDP, a health worker brings ART and clinical services to a community site where 10–50 PLHIV gather. Both models have demonstrated enhanced access to HIV care and psychosocial support in populations outside of refugee settlements [28–31]. Biannual comprehensive clinical exams and laboratory testing remain required in both models. In Uganda, high self-reported ART adherence (89%) has been found among CCLAD and CDDP members [32].

With the roll-out of the November 2022 Consolidated Guidelines for the Prevention and Treatment of HIV and AIDS in Uganda, community ART delivery eligibility was expanded from stable clients to all PLHIV in Uganda [33]. Though limited evidence exists on the effectiveness of this evidence-based differentiated service delivery model for persons newly diagnosed with HIV, initial findings are encouraging. The Delivery Optimization of ART (DOART) randomized trial, which included participants not on ART with a detectable viral load, showed increased viral suppression among participants receiving community-based ART initiation and ART refills through mobile vans compared with clinic-based ART [34]. In South Africa, viral suppression improved after the introduction of a community ART group intervention for clients with elevated viral loads [35].

There is a lack of evidence on community ART for persons newly diagnosed with HIV in the humanitarian context. A pilot study assessing the feasibility of community ART conducted in 2020 among 92 PLHIV in Nakivale Refugee Settlement in Uganda found high acceptability in this context, with over 80% of enrolled individuals willing to participate [36]. Community ART delivery participants appreciated the model’s convenience and the social support and community building it fostered. In the pilot study, group participation over time, however, was challenged by member mobility and fluctuating levels of commitment and reliability. To date, no randomized trials have been conducted on community ART delivery for persons newly diagnosed with HIV in a humanitarian context, and research is needed to assess long-term outcomes and community ART delivery scalability for refugee populations.

The objective of the “Head StART” cluster randomized trial is to evaluate the expansion of community ART delivery to people newly diagnosed with HIV in refugee settlements in Uganda, examining its effectiveness, implementation, and implications for health system costs.

## Materials and methods

### Study design

The Head StART study is a 5-year hybrid type II cluster randomized controlled trial assessing the clinical effectiveness and implementation of community ART delivery, an implementation strategy aimed at enhancing the uptake of ART – the evidence-based treatment for HIV. A cluster design was selected because it reflects the real-world delivery of community-based interventions, minimizes the risk of contamination between study arms, and enables the evaluation of contextual and implementation factors that operate at the health facility.

The Head StART study will investigate 1) the effectiveness of community ART delivery compared to facility-based care in achieving HIV viral suppression among people newly diagnosed with HIV in refugee settlements; 2) the implementation of community ART delivery for people newly diagnosed with HIV in refugee settlements and the impact of contextual factors on study outcomes; and 3) the programmatic cost and budget impact of implementing community ART delivery for people newly diagnosed with HIV in refugee settlements.

**Fig 1:**
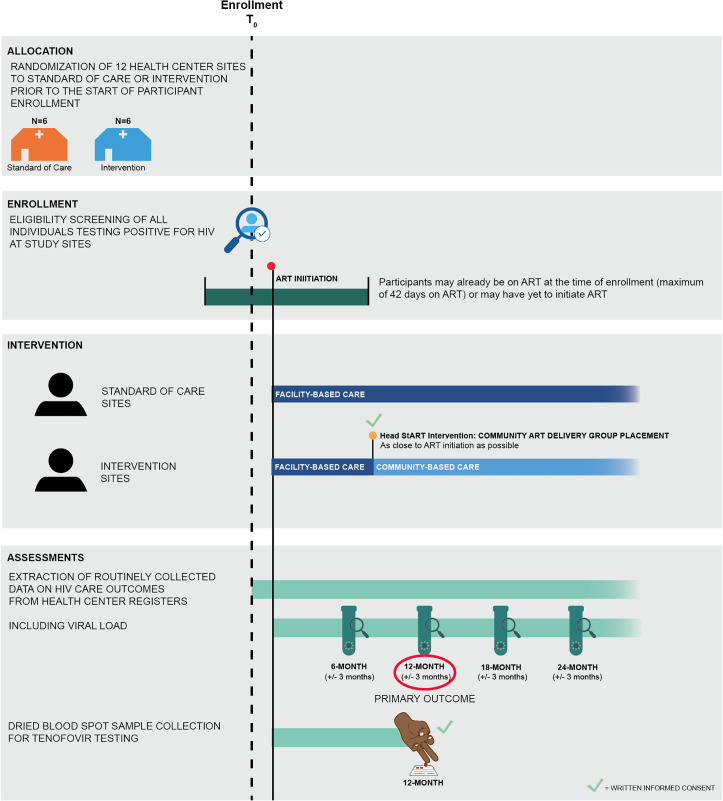
Study overview: schedule of enrollment, interventions, and assessments.

In brief, 12 health centers (clusters) in refugee settlements in Uganda were allocated to either intervention or standard of care (SoC) (N = 6 clusters per arm) using a stratified randomization process (Fig 1). At intervention sites, people newly diagnosed with HIV are offered participation in community ART delivery, while at SoC sites, people newly diagnosed with HIV are offered standard facility-based care. Participants are followed over time, and routinely collected data on HIV care outcomes are extracted from clinic registers. The primary study outcome compares the proportion of newly diagnosed clients who are virally suppressed at 12 months post-ART initiation between intervention and SoC sites. Implementation will be evaluated through the lens of the RE-AIM framework [37]. Data extracted from clinic records, qualitative data from interviews with participants, health workers, and policy leaders, and data collected in direct observations will be used to assess Head StART reach, effectiveness, adoption, implementation, and maintenance across study sites. Intervention costs will be estimated through micro-costing, time and motion observations, and costing interviews. Costs will serve as inputs to a budget impact analysis.

While individuals newly diagnosed with HIV in Uganda have recently become eligible for community ART delivery, the Uganda MoH has approved the delay of community ART delivery implementation for people newly diagnosed with HIV at SoC sites for the duration of the Head StART study.

### Study sites

In September 2022, twelve health centers in five refugee settlements in Uganda (see S2 Appendix) were selected as study sites from the list of health centers in refugee settlements where HIV services were supported by the study partner, Medical Teams International (MTI). To increase generalizability of results and gain insight into the implementation of Head StART in different environments, diversity in geographical location (and by extension sociocultural practices), HIV prevalence, and health center care level was prioritized.

All selected study sites offer free comprehensive HIV services, including HIV testing and ART treatment, to refugees and Ugandan nationals who live within or close to the settlement boundaries. The population served varies by site, with some health centers accessed primarily by refugees and others predominantly by nationals. The sites are located in midwestern and southwestern Uganda, where the majority of refugees come from the Democratic Republic of the Congo, Burundi, Rwanda, and Somalia. The predominant languages spoken in these regions include Kiswahili, Kinyarwanda, Runyankore, and Somali.

### Randomization

**Fig 2:**
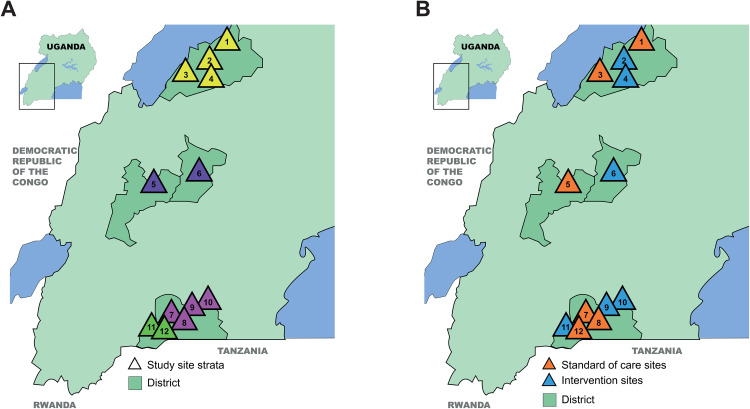
Location of Head StART study sites. **Original Fig by the authors.** 1: Kyangwali Health Center IV; 2: Maratatu B Health Center III; 3: Rwenyawawa Health Center III; 4: Kasonga Health Center II; 5: Rwamwanja Health Center IV; 6: Bujubuli Health Center IV; 7: Kibengo Health Center III; 8: Juru Health Center III; 9: Nakivale Health Center III; 10: Rubondo Health Center III; 11: Nshungyezi Health Center III; 12: Rulongo Health Center III.

A 4-2-2-4 stratified randomization approach was used to allocate the twelve health center sites to intervention and SoC arms; prior to the start of participant enrollment, sites were categorized into four strata by settlement and/or region (Fig 2A). Randomization occurred within these strata to ensure balance between study arms in terms of key site characteristics. Randomization and allocation of sites were performed publicly during the Head StART study inception meeting in Kampala, Uganda, in October 2023, by inviting representatives from key research partners to blindly draw half the entries for each stratum from a bowl containing the site names. Sites that were drawn were allocated to the intervention, and the remaining sites were assigned to SoC (Fig 2B). Enrollment projections were available based on the number of new HIV diagnoses at each health center in the first two quarters of 2023, obtained from MTI program data. Randomization was constrained to reject any result in which the projected number of enrolled participants in one arm was more than twice the number in the other study arm, but this did not occur.

### Study procedures

#### Staff recruitment and training.

Thirteen research assistants were recruited, including from refugee settlements, to be stationed at the 12 study sites and perform data collection activities. In October 2023, a multi-day study inception meeting was held in Kampala, Uganda, during which research assistants and health workers supporting HIV services at study sites were introduced to the Head StART study. Topics covered included ART initiation, relevant Health Management Information System (HMIS) forms, differentiated service delivery, study procedures, participant eligibility, the informed consent process, adverse event monitoring and reporting, and data entry on electronic tablets. All research assistants completed the required training on Human Subjects Protection and Good Clinical Practice. Follow-up and refresher trainings for research assistants are conducted regularly throughout the study, both virtually and during in-person site visits. Health worker staff at study sites and implementing partner research collaborators are informed about study progress during monthly stakeholder meetings.

#### Community advisory boards.

A community advisory board (CAB) was established in each of the five refugee settlements, guided by a Community Liaison Specialist. Community advisory boards consist of 5–6 members and reflect a broad range of community characteristics, including sex, countries of origin, villages in the settlements, and community roles. CABs meet regularly (2–4 times/year). Input and feedback from CAB members are sought on study materials and processes to ensure cultural sensitivity. CAB members are updated on study developments, and their perspectives and advice are solicited regarding any challenges that arise.

#### Participant identification and enrollment.

Study enrollment will span three years and involves reviewing the clinic records of all individuals testing positive for HIV at study sites to determine study eligibility. Individuals who are 1) ≥18 years of age or an emancipated minor (individual <18 years who is pregnant, married, has a child, or is self-sufficient) or a mature minor (individual 14–17 years of age with a drug or alcohol dependency or a sexually transmitted infection) and 2) newly diagnosed with HIV (<6 months prior), not already known to be HIV positive at the time of testing, and not on ART for more than 6 weeks (42 days) at the time of screening are enrolled in the study. Pregnant and breastfeeding women (who receive HIV care through antenatal care and do not participate in community ART delivery), individuals deemed by a clinician to require facility-based care secondary to medical need, and individuals concurrently enrolled in another biomedical clinical trial are excluded from study participation. Each enrolled participant is assigned a unique study identification number.

#### Study procedures.

Study participation entails longitudinal monitoring of participants’ HIV care outcomes through the abstraction of routinely collected HIV data from clinic registers (Fig 2, S3 Appendix). Additionally, study participants at intervention sites are contacted and offered community ART delivery, i.e., the Head StART intervention. To be eligible for the Head StART intervention, participants must speak one of the five study languages (Kiswahili, Runyankore, Kinyarwanda, Somali, or English) and be willing and able to comply with study procedures.

Study participants who consent to the Head StART intervention are assigned to a community ART delivery group, with individuals being initiated into groups as close to their date of diagnosis as possible. The community ART delivery group is either a CCLAD group or a CDDP group, based on the participant’s preference, group availability, and group proximity to their residence. Intervention participants join existing community ART delivery groups whenever possible. In instances where a new group is needed due to group size or geographic location, a new group is formed.

Community ART delivery is implemented following the Uganda MoH Implementation Guide for Differentiated Service Delivery Models of HIV Services in Uganda (Table 1) [20]. Annual comprehensive clinical evaluations and viral load testing occur either at the health center (CCLAD model) or in the community (CDDP model). Intervention participants in CCLAD and CDDP models retain the ability to visit the health center at any time, including when they are unwell, have questions for the health worker, if laboratory testing is needed, or if requested by the health worker. Intervention participants may withdraw from participation in the Head StART intervention at any time. Those who elect to withdraw are invited to participate in a brief exit interview. Withdrawing from the intervention does not impact study participation or data abstraction on HIV care outcomes. Intervention participants who become pregnant, non-suppressed, or develop a medical condition that requires management at the health facility will be required to return to facility-based care until they are once again deemed eligible by the clinician for community-based care. Study participants at intervention sites who decline community ART delivery receive facility-based care per Uganda MoH protocols, which could include facility-based individual management, facility-based groups, or fast-track drug refill [33].

**Table 1:** HIV care models in Uganda.

	Facility-based care	Community ART delivery	
	Facility-based individual management	Community client-led ART delivery (CCLAD)	Community Drug Distribution Point (CDDP)
Group size	Not applicable	3-6	10-50
ART refill	Individuals visit the health facility where they receive their ART medication from a health worker	Group members take turns picking up ART at the health facility and distribute ART among the other group members in the community. Drug pickup consists of: A pre-drug pick-up meeting during which client books are collected by the group representative and health/nutrition/pregnancy/family planning/TB status/pill count are assessed A visit to the health facility by the group representative to pick up ART for the group A post-drug pick-up meeting during which the representative distributes ART to the group	Health workers bring ART and other services (tuberculosis drugs, tuberculosis screening, family planning, pregnancy screening, nutrition assessment, opportunistic infection screening and management) to a distribution point in the community
Viral load monitoring	At 6 months post-ART initiation, 12 months post-ART initiation, and annually thereafter during comprehensive clinical evaluation visit at the facility	At 6 months post-ART initiation, 12 months post-ART initiation, and annually thereafter during comprehensive clinical evaluation visit at the facility	At 6 months post-ART initiation, 12 months post-ART initiation, and annually thereafter at the CDDP location

At SoC sites, study participants receive facility-based care per Uganda MoH protocols. Participants at SoC sites who have completed 12 months on ART and are considered “stable” can, at that point in time, receive community ART delivery if they are willing.

At both intervention and SoC sites, viral load testing is conducted 6 months post-ART initiation, 12 months post-ART initiation, and annually thereafter, in accordance with the Uganda MoH guidelines. Per current practice, viral load samples, collected through finger prick or blood draw, are transported to the Central Public Health Laboratories for analysis. Plasma HIV RNA levels are quantified using one of the available validated platforms at the lab (Abbott m2000 Real-Time HIV-1 or Roche COBAS® Ampli Prep v 2.0 assay) [38]. As part of the Head StART study, at the time of routine 12-month viral load testing, participants are invited to provide an additional blood sample to create a dried blood spot (DBS) for tenofovir diphosphate (TFV-DP) concentration measurement. For a random sample of participants whose 12-month viral load is non-suppressed, TFV-DP levels will be quantified to evaluate poor ART adherence as a possible mechanism for non-suppression. Selected DBS samples will be sent to the DAIDS-certified Pharmacology Lab at the University of Chiang Mai (ISO:15189 certified; Lab Director, Tim R. Cressey), where TFV-DP levels will be assessed using validated liquid chromatography with tandem mass spectrometry [39,40].

#### Participant follow-up.

Participants are followed from enrollment through the end of the 5-year study period or until the participant reaches a study endpoint, including death or transfer of HIV care to a non-study site. Participant follow-up consists of routine clinic visits and (for intervention participants) community ART visits.

#### Blinding.

The study is unblinded. Blinding of study staff and participants is not feasible given the distinct protocols of the two study arms.

#### Data collection.

*Head StART effectiveness:* At intervention and SoC sites, routinely collected data are abstracted by research assistants from written HIV clinic registers, laboratory registers, pharmacy registers, and, if available at study sites, from the electronic medical record system for all study participants. Research assistants enter abstracted data directly into Mobile REDCap, a secure web-based platform with offline capacity, which is installed on passcode-protected, encrypted electronic devices. Collected data is routinely pushed by research assistants from the electronic devices to the secure online REDCap platform. Collected variables include demographic characteristics, ART initiation date, ART regimen, dates and results of viral load tests, dates of ART medication pick-ups, and HIV clinic/community ART group visit dates. Data captured in the online REDCap platform is verified with a target frequency of once per week to facilitate timely and reliable data quality control and assurance. Additionally, quarterly site visits are performed to allow for independent comparison of data entered in REDCap with written registers.

To optimize the routine collection of viral load samples at 12 months post–ART initiation (primary study outcome), outreach activities—including follow-up phone calls and community visits—are conducted by study staff, as resources permit, to trace participants who did not return for their 12-month viral load test and encourage completion of this assessment.

*Head StART implementation:* At intervention sites, semi-structured in-depth interviews are conducted with a purposive sample of study participants (N = 70). Participants are sampled to include both those who agreed (N = 60) and those who declined intervention participation (N = 10), enrolling refugees from different countries of origin and Ugandan nationals, and participants who are virally suppressed and unsuppressed at 12 months post-ART initiation. For selected participants receiving the Head StART intervention, interviews are conducted at 0 (N = 20), 6 (N = 20), and 12 months (N = 20) from ART initiation to understand the influence of Head StART on HIV care engagement over time. Additionally, interviews will be conducted with health center staff and policy leaders (N = 10).

All interviews are scheduled at a time and location convenient for the participant with the goal of maximizing confidentiality. A multilingual research assistant trained in qualitative techniques conducts a private interview of approximately one hour in length in the participant’s preferred study language with the assistance of an interpreter when necessary. Interviews follow a semi-structured interview guide informed by the RE-AIM framework [37] and explore factors influencing Head StART participation (reach), benefits and any negative effects of Head StART (effectiveness), the extent to which Head StART components are embraced by staff (adoption), reasons for practice variations/adaptations in Head StART implementation (implementation), influence of Head StART on intervention participants over time (maintenance), and changes in Head StART implementation by staff throughout the study (maintenance). Interviews are audio-recorded with permission. Participants are reimbursed 20,000 Uganda shillings (~5.63 US dollars) for their time and transportation costs.

Direct observations are conducted to assess Head StART adoption, implementation, and maintenance. Using structured checklists informed by the MoH’s Implementation Guide for Differentiated Service Delivery Models of HIV and TB Services in Uganda [41], research assistants at intervention sites evaluate the Head StART components delivered to participants. Additionally, research assistants provide descriptive notes on specific aspects of observed activities guided by prompts. Observations occur monthly on purposively selected days.

Any modifications or adaptations to the Head StART intervention or to HIV service delivery more broadly with the potential to influence study outcomes that are brought to the attention of study staff are documented in accordance with the Framework for Reporting Adaptations and Modifications to Evidence-based Implementation Strategies (FRAME-IS) [42].

*Head StART costing:* Micro-costing, costing interviews with staff, and time and motion observations are conducted to estimate the cost of implementing Head StART as part of routine HIV care in refugee settlements. Costs of the intervention and SoC activities are collected from the Head StART trial using the payer (Uganda MoH) perspective. Standardized Excel cost menus are utilized to collect intervention costs, including start-up costs (e.g., HIV clinic worker training), human resources, supplies, and participant transport reimbursement. Interviews are conducted with staff working in the HIV clinic to assess daily responsibilities associated with implementing Head StART. Time and motion observations are conducted in a representative subset of intervention sites to assess variation in Head StART implementation by setting (e.g., clinic volume, clinic population). Research time (e.g., informed consent) is removed from programmatic costs.

### Outcome measures

The primary study outcome will be assessed at the cluster level at the end of the study and consists of the proportion of participants who are virally suppressed (<1,000 copies/mL, consistent with Uganda MoH definitions at the time of study design [43]), 12 months (+/- 3 months) post-ART initiation. Secondary outcomes are the proportion of participants who are virally suppressed at 6 months (+/- 3 months) post-ART initiation, and TFV-DP concentrations at 12 months (+/- 3 months) post-ART initiation among virally unsuppressed participants [44,45].

### Sample size and power calculation

It is anticipated that at SoC sites, at 12 months post-ART initiation, 60% of participants will be virally suppressed (<1000 copies/mL). Community ART delivery participation is hypothesized to be associated with a 20%-point increase in viral suppression 12 months post-ART initiation. To account for less than perfect participation (75% participation, a conservative estimate based on pilot data), an average community ART delivery effect size of 15%-points is assumed. Assuming an inter-cluster correlation of 0.02, power of 80%, and 5% margin of error (2-sided), a sample size of 1,360 participants per study arm (total 2,720) is required to ensure adequate power to address the study’s primary hypothesis that community ART delivery increases viral suppression at 12 months post-ART initiation by 15% compared to standard of care. To account for loss to follow-up (~15% attrition), up to 1,560 participants will be enrolled per arm (up to 3,120 total).

### Statistical methods and analysis

#### Head StART effectiveness.

The primary analysis will estimate the difference in the prevalence of viral suppression 12 months post-ART initiation, comparing intervention and SoC clusters using a two-stage approach recommended for cluster randomized trials with a small number of clusters per group [46]. Prevalence ratios will be calculated comparing the proportion of those with viral suppression in the two study arms by taking the geometric mean of the prevalence ratio observed in each of the four health center strata and constructing a 95% confidence interval using a normal approximation [46]. To help overcome participation bias, an intention-to-treat approach will be taken. For those instances when the 12-month viral load outcome is missing for a participant, viral non-suppression will be assumed to fit the most conservative models. Missingness patterns will be assessed and sensitivity analyses of the primary outcome will be performed, including a sensitivity analysis in which missing data will be imputed using multiple imputation and an analysis in which ~30% viral suppression will be assumed among those without viral load test results in line with prior research in sub-Saharan Africa that demonstrated that 71% of participants lost to follow-up had high viremia (>1000 copies/mL) [47]. Sensitivity analyses will also include using more stringent thresholds for viral suppression (<400 copies/mL and <200 copies/mL) [48–51]. Descriptive summaries of the quantitative measures will be presented, and descriptive statistics will be used to compare categorical and continuous variables between intervention and SoC arms.

Secondary outcomes that will be examined include the estimated difference in the prevalence of viral suppression at 6 months post–ART initiation between intervention and SoC arms, as well as the quantification of TFV-DP concentrations (mean, median, and interquartile range) for each study arm. Linear regression models will be used to assess differences in TFV-DP levels between the intervention and SoC sites. Additionally, TFV-DP concentrations will be categorized as low, medium, or high to represent varying levels of ART adherence. The distribution of adherence levels will be compared between study arms using ordinal logistic regression models, accounting for clustering by site.

#### Head StART implementation.

Qualitative interviews will be transcribed verbatim from audio-recordings. For interviews conducted with the assistance of an interpreter, only the English questions and responses will be transcribed. For interviews conducted by research assistants in other study languages, audio-recordings will be translated into English and transcribed. Qualitative software (e.g., Dedoose or Atlas.ti) will be used to store and analyze in-depth interview and direct observation data. Data collection and analysis will occur in parallel. Two coders will read a randomly selected interview to identify general themes using the RE-AIM domains to initially organize the data. A content analysis approach will then be used to identify additional inductive codes under each RE-AIM domain to demonstrate determinants influencing Reach, Effectiveness, Adoption, Implementation, and Maintenance of the Head StART intervention [37]. Based on these codes, the coders will create a preliminary codebook, discuss initial findings, and resolve discrepancies. The codebook will be developed iteratively by coding an additional subset of transcripts together. The revised codebook will be applied to the remaining interviews. It is expected that the envisioned sample size of 80 interviews will yield sufficient informational power to evaluate Head StART implementation [52], but adequacy of the sample size will be assessed continuously throughout the parallel data collection and analysis process, and additional participants will be recruited as necessary.

For analysis of the direct observation data to understand Head StART adoption, implementation, and maintenance at the health center sites, the coding process will be iterative as changes are expected to occur over time. At the conclusion of the study, key themes will be described, addressing potential variability observed over time and differences by study site.

Through convergence and complementarity, quantitative and qualitative results will be connected, using qualitative data to provide depth of understanding [53–55]. The concurrent collection of quantitative and qualitative data will provide an opportunity for the quantitative results to inform subsequent qualitative data collection [56]. Similarly, quantitative data will be used to evaluate assertions arising from the qualitative data.

#### Head StART costing and budget impact analysis.

Costs per client will be estimated as the total annual program costs divided by the number of clients. Costs and outcome data from the trial will be combined in an Excel-based Markov model parameterized to reflect HIV natural history and care engagement of refugees in Uganda. The model will be used to project the annual costs and budget impact of implementing the Head StART intervention, incorporating the eligible refugee population size, current and future intervention mix (SoC and Head StART), and total health expenditures. We will utilize published estimates of ART drug costs, laboratory monitoring, and HIV-related hospitalizations to estimate program costs incurred and averted by Head StART implementation. Following guidelines established by The Professional Society for Health Economics and Outcomes Research [57], a 5-year time horizon will be used. Two scenarios will be modelled: 1) Head StART implementation and 2) SoC. We will estimate HIV-related deaths and hospitalizations averted in Head StART versus SoC. The budget impact of Head StART will be assessed by accounting for costs incurred and averted by the intervention as the difference in cost between the intervention and SoC scenario. Univariate and probabilistic sensitivity analyses will be conducted to assess the influence of model assumptions. Monte Carlo simulation will be used to generate uncertainty intervals around cost estimates. Variations in budget impact by HIV clinic size, location, and number of clients newly diagnosed with HIV will be assessed. Secondary model outcomes will include viral suppression and HIV deaths averted in the Head StART scenario compared to SoC over the 5-year time horizon.

### Ethical approval

This trial was approved by the Makerere University Infectious Diseases Institute Research Ethics Committee (IDI-REC-2022-20) and the University of Washington Human Subjects Institutional Review Board committee (STUDY00016473). In accordance with national guidelines in Uganda, approval was also obtained from the Uganda National Council for Science and Technology (HS2935ES). Support was granted from the Refugee Desk Officer in the Uganda Office of the Prime Minister and the AIDS Control Program Manager in the Uganda MoH. The trial is registered at ClinicalTrials.gov (NCT06126913) [1].

The obtained ethical approval includes a waiver of consent for the extraction of participant data routinely collected as part of HIV care. Written informed consent is obtained for study activities, including participation in community ART delivery, DBS sample collection for TFV-DP testing, sample storage, and in-depth qualitative interviews with PLHIV. Verbal consent is obtained for in-depth qualitative interviews with clinic staff and policy leaders, costing interviews, and direct observations. As part of the informed consent process, participants are assured that their data will be kept confidential and that their decision to participate will not affect their access to or quality of HIV care services, nor will it impact the employment security of staff. Participants are reminded that they are not required to take part in any study activities and may elect to withdraw at any time. Written informed consent will be collected either on hardcopy consent forms or by having participants sign an electronic version of the consent form in REDCap on an electronic tablet. For illiterate participants, a literate impartial witness will be present throughout the consent process, and a participant mark or thumbprint is obtained on hard-copy consent forms.

A number of study protocol revisions were made following initial ethical approval. All revisions were reviewed and approved by the Makerere University Infectious Diseases Institute Research Ethics Committee and the University of Washington Human Subjects Institutional Review Board. These revisions included the change from matched-pair to stratified-pair randomization to optimize statistical efficiency, accompanied by updated sample size calculations reflecting the revised approach and anticipated loss to follow-up. Eligibility criteria were revised to enhance inclusivity and clarity; specifically, enrollment was expanded to include mature and emancipated minors, and exclusion criteria were narrowed from excluding all participants with comorbidities or higher WHO clinical stages to excluding only those judged by clinicians to require facility-based care. Additionally, it was clarified that clients testing HIV-positive in the prior six months were eligible only if not previously known to be HIV-positive. The allowable window for viral load and tenofovir level outcomes was extended from ±2 months to ±3 months and language regarding tenofovir testing and DBS sample collection, testing, and storage was clarified. Language regarding Data Monitoring Committee composition was revised and reporting requirements were revised based on feedback from the Data Monitoring Committee and the Institutional Review Board. Finally, plans for direct observation data collection were updated.

### Adverse event and data monitoring

All adverse events occurring during the study will be reviewed to assess potential study-relatedness, considering factors such as timing, alternative explanations, and background incidence. Unanticipated adverse events—those that are unexpected, pose increased risk, and are reasonably related to study participation—along with all serious adverse events, will be reported in accordance with institutional review board requirements and local and international regulations. A Data Monitoring Committee, comprising a biostatistician, an HIV clinician, and a clinical trials researcher with experience in sub-Saharan Africa, will convene every six months to review study implementation, data quality, outcomes, and safety events.

### Trial status

Participant recruitment and enrollment began on January 15th, 2024. At the time of submission, participant enrollment and data collection are ongoing.

### Reporting

The results of this study will be reported following the Consolidated Standards of Reporting Trials (CONSORT) 2025 [58].

### Inclusivity in global research

Additional information regarding the ethical, cultural, and scientific considerations specific to inclusivity in global research is included in the Supporting Information (S4 File).

## Discussion

The Head StART study is a pragmatic cluster-randomized trial evaluating the effectiveness, implementation, and budget impact of community ART delivery for people newly diagnosed with HIV in refugee settlements in Uganda. The primary objective is to determine whether this differentiated service delivery model can improve care engagement and clinical outcomes, with the primary outcome, viral suppression, serving as a proxy indicator of consistent ART use and retention in care.

It is hypothesized that community-based care offers important benefits for individuals newly diagnosed with HIV, who often face stigma, limited social support, and competing survival needs. Generating evidence on the safety and effectiveness of this approach for persons newly initiating ART is particularly urgent due to the rollout of Uganda’s 2022 Consolidated HIV Guidelines, which made community ART delivery available to all persons with HIV. Community ART delivery is being implemented at scale and safety concerns remain as reduced facility contact during this early, vulnerable period could contribute to loss to follow-up or missed detection of ART-related side effects and immune reconstitution inflammatory syndrome.

To strengthen real-world HIV service delivery applicability, in addition to evaluating community ART delivery effectiveness, the Head StART trial includes a robust implementation science component designed to assess how the intervention is implemented, adapted, and experienced in the resource-constrained context of refugee settlements. This comprehensive scope enables the study to not only capture whether the intervention works, but how, why, and under what conditions it is most effective. Detailed data are collected on fidelity, adaptations, and implementation quality, as well as contextual factors that may influence outcomes. The study will also assess the feasibility and sustainability of the intervention over time, providing insight into long-term program viability. Qualitative interviews with individuals who accept and decline the intervention at various points in their care trajectory will help identify subpopulations for whom community ART delivery is most acceptable and effective.

Additionally, the budget impact analysis included in the Head StART study will generate essential data for the Uganda MoH to inform resource allocation and planning, as community ART delivery—while designed to alleviate the health system burden by decentralizing care—may require additional investments in refugee settlements related to transportation logistics and intensified outreach efforts.

A number of study limitations warrant consideration. First, while the intention-to-treat approach minimizes bias and enhances real-world applicability, it may, when combined with logistical challenges in assigning clients to community ART groups, dilute the intervention effect and potentially obscure the true benefits of community ART delivery on viral suppression. To address this, research assistants work closely with HIV counsellors to facilitate the timely placement of participants into community ART groups as close to ART initiation as possible. Study teams also share best practices across sites to promote consistent implementation and optimize group formation.

Second, the study’s reliance on routine healthcare practices, including HIV testing, ART initiation, and viral load monitoring for participant enrollment and outcome ascertainment, while enhancing external validity, introduces vulnerability to system-level disruptions. Interruptions in national health service delivery, such as stockouts, human resource shortages, or policy shifts, could affect data availability and the timely delivery of services essential to the study. To mitigate this risk, we work closely with the Uganda MoH and implementing partners to align study activities with national systems and enable early detection of service disruptions.

Finally, high mobility of the refugee population, including relocation to other settlements and extended travel to countries of origin, may pose challenges for follow-up and result in missing data for the primary study outcome. In anticipation of this challenge, we incorporated a 15% over-enrollment and allocated resources for enhanced outreach to support 12-month viral load sample collection. However, if attrition exceeds these projections, the completeness of primary outcome data and study power may be affected.

## Conclusion

The Head StART cluster randomized controlled trial will provide important evidence on the effectiveness, implementation, and cost of community ART delivery for individuals newly diagnosed with HIV in refugee settlements in Uganda – a population at elevated risk of viral non-suppression and attrition from HIV care. With community ART delivery recently introduced and scaled up for this group, timely findings will be pivotal to inform national HIV policy and enhance care for refugees, whose equitable access to HIV services has been recognized as a priority by international health and humanitarian agencies. The study addresses a significant gap in understanding how differentiated service delivery functions in humanitarian settings.

## Supporting information

S1 File SPIRIT checklist. (PDF)

S2 Appendix MTI programmatic data for Head StART study sites Q1 & Q2 2023. (PDF)

S3 Appendix Study protocol. (PDF)

S4 File Inclusivity in global research checklist. (DOCX)
