# Supplementary material for: Protocol for Head StART: A hybrid type II cluster randomized controlled trial evaluating community ART delivery for people newly diagnosed with HIV in refugee settlements in Uganda
Source: PLoS One. 2026 Feb 27;21(2):e0340916. doi: 10.1371/journal.pone.0340916 (PMC12948099; doi:10.1371/journal.pone.0340916)
Supplement: S2 Appendix — (PDF) [file pone.0340916.s002.pdf]

MTI programmatic data for Head StART study sites Q1 & Q2 2023

Region	Refugee Settlement	Site	Consultations/ month	People tested for HIV	HIV diagnoses	Positivity rate
Southwest	Nakivale	Nakivale Health Center (HC) III	9,202	2,700	68	2.52%
		Juru HC III	3,010	1,057	35	3.31%
		Kibengo HC III	1,804	913	19	2.08%
		Rubondo HC III	5,635	1,387	17	1.23%
	Oruchinga	Rulongo HC II	1,579	906	17	1.88%
		Nshungyezi HC III	1,948	1,865	39	2.09%
Midwest	Kyaka II	Bujubuli HC IV	18,225	3,559	85	2.39%
	Rwamwanja	Rwamwanja HC IV	12,147	4,463	66	1.48%
	Kyangwali	Kyangwali HC IV	3,292	5,045	87	1.72%
		Kasonga HC II	1,808	1,177	7	0.59%
		Maratatu B HC III	3,146	2,527	34	1.35%
		Rwenyawawa HC III	2,412	2,597	20	0.77%
