# Supplementary material for: Protocol for Head StART: A hybrid type II cluster randomized controlled trial evaluating community ART delivery for people newly diagnosed with HIV in refugee settlements in Uganda
Source: PLoS One. 2026 Feb 27;21(2):e0340916. doi: 10.1371/journal.pone.0340916 (PMC12948099; doi:10.1371/journal.pone.0340916)
Supplement: S3 Appendix — (PDF) [file pone.0340916.s003.pdf]

TITLE:

Achieving HIV viral suppression in refugee settlements in Uganda with Head StART: a cluster randomized trial evaluating the effectiveness of community ART delivery for people newly diagnosed with HIV

Area: HIV/refugees

Type: Type III implementation/effectiveness cluster- randomized controlled trial

List of investigators and co-investigators:

Name	Title/Role	Address/Institution	Phone number	E-mail
Kelli O'Laughlin	PI	UW	+ 1 (503) 705-5354	kolaugh@uw.edu
Timothy Muwonge	Site-PI	IDI	+ 256 752 811 000	tmuwonge@idi.co.ug
Monisha Sharma	Co-I	UW	+ 1 (617) 360-1625	msharma1@uw.edu
Melissa Mugambi	Co-I	UW	+ 1 (206) 616-9711	mugambi@uw.edu
Paul Drain	Co-I	UW	+ 1 (206) 306-3066	pkdrain@uw.edu
Agnes Kiragga	Co-I	IDI Makerere	+ 256 772 512 026	akiragga@idi.co.ug
Andrew Mujugira	Co-I	IDI Makerere/UW	+ 1 (206) 489-8538	mujugira@uw.edu

DOCUMENT HISTORY

Document	Version Date	Summary of Changes	PI Signature
Amendment 3	Version 4, 8 December 2023	1. The health implementing partner name, "Medical Team International" was deleted or exchanged for "health implementing partner" as there may be a different health	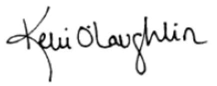

		implementing partner in the future of this study.<br>2. Definitions were added to “mature minor” and “emancipated minor” in the inclusion criteria section (4.1.1).<br>3. Section 4.2.1 was revised as we do not plan to unenroll people mid-study but to instead follow their clinical outcomes after they are returned from community-based care back to facility-based care for any reason (e.g., pregnancy, advanced HIV disease state)<br>4. Updates were made following the first convening of the Data Monitoring Committee to better align adverse event reporting requirements given this is not a drug trial but a trial to assess a community-based ART delivery strategy.<br>5. Added details to indicate that the Tenofovir drug levels will be tested using dried blood spots.	
Amendment 2	Version 3, 2 October 2023	1. Inclusion criteria were expanded to include mature minors.<br>2. Section 13 regarding post-trial access information was inserted.	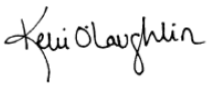
Amendment 1	Version 2, 12 September 2023	1. Removed Co-I Deepa Rao.<br>2. Updated randomization approach from matched pair to stratified randomization.	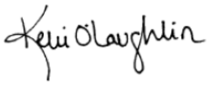

		3. Updated sample size calculations, new total N=2,720 (1,360 per arm) recruiting up to N=3,120 (1,560 per arm) to account for attrition.<br>4. Adapted inclusion and exclusion criteria to include emancipated minors, limited the exclusion of those with comorbidities and higher WHO clinical stage to those participants for whom clinicians deem facility-based care necessary, clarified that index clients testing positive should only be included if not already known to be HIV positive.<br>5. Edited details regarding tenofovir sample processing.<br>6. Changed composition requirements for data and safety monitoring committee.<br>7. The Capacity Building section was revised to describe personnel involved without specifying their percent effort on the grant.	
Original protocol	Version 1, 2 December 2022	N/A	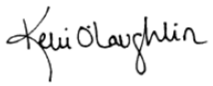

PROTOCOL SUMMARY

Background/Rationale: Refugees face barriers to Human Immunodeficiency Virus (HIV) care engagement, including long distances to clinics, high transportation costs, inclement weather that limits travel, and scarce livelihood opportunities [1-3]. In addition, refugees suffer from disrupted social networks, which are strongly associated with HIV care engagement [1, 3]. Uganda has nearly 1.6 million refugees, one of the largest refugee populations in the world [4]. In our prior studies from Nakivale Refugee Settlement, only 54% of 276 and 74% of 219 persons with HIV were linked to HIV care [2, 3]. Barriers to care for refugees impede progress toward UNAIDS 95-95-95 targets [5].

Community antiretroviral therapy (ART) delivery is a differentiated care model that fosters social support and reduces time and transportation barriers yielding improved engagement in HIV care [6-11]. In Uganda, to participate in community ART delivery, clients must be "stable" in care (> 1 year on ART and viral load < 1,000 copies/mL) [12, 13]. Therefore, persons newly diagnosed with HIV are not eligible for community ART delivery. Community ART delivery may benefit persons newly diagnosed with HIV in refugee settlements by bolstering social support and by bringing ART closer to individuals living in these expansive rural settlements. In our pilot in Nakivale Refugee Settlement in Uganda, community client-led ART delivery implemented shortly after HIV diagnosis was feasible, acceptable, and safe. Of participants with a viral load result after 6 months, 87% were suppressed (<1,000 copies/mL) [13]. Participants reported lower transport costs, stigma, stress, and improved social support, access to ART, and adherence. The primary objective of this research is to evaluate the effectiveness of expanding community ART delivery to clients newly diagnosed with HIV.

Objectives and endpoints:

Aim 1: To evaluate the effectiveness of "Head StART," the expansion of community ART delivery to people newly diagnosed with HIV, in achieving HIV viral suppression in refugee settlements in Uganda. Approach: We will conduct a stratified 2-arm cluster randomized controlled trial at 12 health centers in refugee settlements in Uganda, in which people diagnosed with HIV within the prior 6 months will be offered community ART delivery. Outcomes for health centers randomized to Head StART will be compared to those at standard care sites. The primary outcome is the proportion of persons newly diagnosed with HIV who are virally suppressed at 12 months. Secondary outcomes are viral suppression at 6 months and ART adherence at 12 months (assessed by determining tenofovir diphosphate concentrations in dried blood spots among those virally unsuppressed). *Hypothesis**:** Head StART health centers will have a higher proportion of clients with viral suppression than standard care.*

Aim 2: To assess Head StART implementation across refugee settlement sites to understand the impact of contextual factors on study outcomes. Approach: Using the RE-AIM implementation science framework, we will evaluate Head StART in terms of its: **1)** **R**each: Proportion of eligible individuals at intervention sites participating in Head StART (quantitative) and factors influencing participation (qualitative). **2)** **E**ffectiveness: Perceptions of Head StART benefits and contributions to ART adherence/viral suppression (qualitative). **3)** **A**doption: Perspectives of participants and staff on Head StART adoption (qualitative). **4)** **I**mplementation: Adherence to Head StART core components and coverage of intervention (quantitative) and quality of intervention delivery, variation in implementation, and reasons for adaptations (qualitative). **5)** **M**aintenance: Sustained participant effects of Head StART and duration of intervention delivery (quantitative), influence on participants, and reasons for changes over time (qualitative). *Hypothesis: The Head StART intervention will be most effective in communities with high reach and strong adoption of the intervention.*

Aim 3: To estimate the programmatic cost and budget impact of implementing the Head StART intervention in refugee settlements in Uganda. Approach: We will conduct activity-based costing and time-in-motion observations to estimate the incremental costs of Head StART implementation. We will combine cost with clinical outcome data from Aim 1 into a Markov model to project the health impact (HIV deaths and morbidity averted) and financial costs of the intervention compared to the standard of care and to estimate the 5-

year budget impact of Head StART implementation. *Hypothesis: Head StART can be affordably implemented in refugee settlements in Uganda.*

Study Design: Using a stratified randomization approach, we will randomize health centers (12 total) to intervention sites or standard care and conduct a 2-arm cluster randomized controlled trial with the primary outcome of HIV viral suppression at 12 months among newly diagnosed individuals (Aim 1). We will use the RE-AIM framework to assess the Reach, Effectiveness, Adoption, Implementation, and Maintenance of Head StART across study sites (Aim 2). We will estimate the cost and budget impact of the intervention to inform future policy decisions (Aim 3). This trial will help to evaluate the effectiveness of the Head StART intervention on viral suppression while gathering information on implementation.

Study Treatments: At intervention sites, newly enrolled individuals who consent to participate in the intervention will be assigned to a community ART delivery group (either a community client-led ART delivery [CCLAD] group or a community drug distribution point [CDDP] group). Newly diagnosed individuals (diagnosed in the prior 6 months) at intervention sites will be offered community ART delivery on a rolling basis during enrollment, initiating individuals into groups as close to their date of diagnosis as possible. Newly enrolled individuals will join existing community ART delivery groups. When a new group is needed based on group size or geographic location, a new group will be formed. At sites randomized to standard care, participants newly diagnosed with HIV will receive facility-based care per Uganda MoH protocols [13].

Statistical design: We will conduct an intention-to-treat analysis of the viral suppression at 12 months post HIV diagnosis (primary outcome; viral suppression defined as VL<1000 copies/ul per Uganda MoH). Our primary analysis will estimate the difference in the prevalence of viral suppression, comparing the intervention and standard care clusters using a two-stage approach. We will calculate prevalence ratios comparing the proportion of those with viral suppression in the two study arms by taking the geometric mean of the prevalence ratio observed in each of the strata and constructing a 95% confidence interval using a normal approximation. If primary outcome data are missing or in cases of loss to follow-up, we will assume individuals are not virally suppressed to fit the most conservative models. We will also conduct sensitivity analyses assuming only 29% of participants lost to follow-up are virally suppressed (Sikazwe et al *PLoS Med* 2019). Secondary outcomes include viral suppression at 6 months and alternative viral suppression thresholds used in recent trials (<400 copies/mL and <200 copies/mL). An additional secondary outcome will include the quantification of intracellular tenofovir diphosphate (TFV-DP) concentrations in dried blood spot samples among those who are virally unsuppressed at 12-months.

Power justification: Viral suppression for individuals accessing services in refugee settlements in sub-Saharan Africa is notably low. We believe that sites in the Head StART intervention will experience an additional 20%-point increase in the proportion of virally suppressed clients at 12 months compared to those not participating in Head StART. To account for less than perfect participation in the intervention at the intervention sites (we estimate 75% participation based on pilot data), we assume an average effective size of 15% points (20% point * 0.75 participation= 15% points) in viral suppression at the intervention sites. Overall, we anticipate 60% viral suppression [<1000 copies/mL at 12 months] at control sites compared to 75% viral suppression at intervention sites. Assuming an inter-cluster correlation of 0.02, power of 80%, and 5% margin of error (2-sided), the required sample size is 1,360 newly diagnosed per arm (total 2,720). To account for the expected loss to follow-up, we will aim to enroll up to 1,560 per arm (up to 3,120 total). Based on the number of clients newly diagnosed with HIV in the study site health centers from July 2021- June 2022, we are confident we will reach our sample size targets over the planned enrollment period.

1. LIST OF ABBREVIATIONS AND DEFINITIONS OF TERMS

**AE**	Adverse event
**ART**	Antiretroviral therapy
**CCLAD**	Community client-led ART delivery
**CDC**	Centers for Disease Control and Prevention
**CDDP**	Community drug distribution point
**CI**	Confidence interval
**CRF**	Case report form
**DAIDS**	National Institute of Allergy and Infectious Diseases Division of AIDS
**DRC**	Democratic Republic of the Congo
**DMC**	Data Monitoring Committee
**GCP**	Good Clinical Practice
**HIV**	Human Immunodeficiency Virus
**IDI**	Infectious Diseases Institute
**IRB**	Institutional Review Board
**LTFU**	Loss to follow-up
**MoH**	Uganda Ministry of Health
**MW**	Midwestern
**NIMH**	National Institute of Mental Health
**OR**	Odds Ratio
**PEPFAR**	United States President's Emergency Plan for AIDS Relief
**PHI**	Patient health information
**PI**	Principal Investigator
**PMTCT**	Prevention of mother-to-child transmission
**PrEP**	Pre-Exposure Prophylaxis
**QC**	Quality control
**RA**	Research assistant
**RCT**	Randomized controlled trial
**RR**	Relative risk
**SAE**	Serious Adverse Event
**SOC**	Standard of care
**SOP**	Statement of purpose
**SW**	Southwestern
**TFV-DP**	Tenofovir diphosphate
**UNCST**	Uganda National Council for Science and Technology
**VL**	Viral load

TABLE OF CONTENTS

1. LIST OF ABBREVIATIONS AND DEFINITIONS OF TERMS 6
1.1. Disease Setting/Patient Population 9
1.2. Background and Rationale 10
2. STUDY OBJECTIVES AND ENDPOINTS 11
2.1. Objectives 11
3. STUDY DESIGN 12
4. SUBJECT SELECTION 12
4.1. Inclusion Criteria 12
4.1.1. Inclusion criteria for Head StART intervention participation (intervention participants) 12
4.1.2. Inclusion criteria for study enrollment (study participants) 13
To be eligible to be enrolled in the Head StART study and monitored over time through data abstraction, subjects at intervention and standard care sites must meet the following inclusion criteria: 13
4.2. Exclusion Criteria 13
4.2.1. Exclusion criteria for study enrollment and Head StART intervention participation 13
4.3. Waiver of consent 13
4.4. Randomization Criteria 13
4.5. *Other* 14
5. TREATMENTS OF SUBJECTS 15
5.1. Allocation to Treatment/Group 15
5.2. Breaking the Blind 15
5.3. Drug Supplies 15
5.3.1. Administration 15
5.3.2. Drug Storage and Drug Accountability 15
5.4. Concomitant Medication(s) 15
6. STUDY PROCEDURES 16
6.1. Screening 16
6.2. Study Period 16
6.3. Follow-up Visit 17
6.4. Subject Withdrawal 17
7. ASSESSMENTS 18
7.1. Efficacy 18

7.2. Implementation assessment 19
7.3. Programmatic cost and budget impact assessment 20
7.4. Pharmacokinetics Assessments 20
7.4.1. Dried blood spot samples for analysis of Tenofovir diphosphate (TFV-DP)20
7.4.2. Justification for PK/other sample shipment 20
7.5. Pregnancy Testing 20
8. ADVERSE EVENT REPORTING 21
8.1. Terminology 21
8.1.1. Non-Medical Adverse Event (Non-Medical AE) 21
8.1.2. Medical Adverse Event (Medical AE) 21
8.1.3. Non-Medical Serious Adverse Event (Non-Medical SAE) 21
8.1.4. Medical Serious Adverse Event (Medical SAE) 21
8.2. Severity Assessment 21
8.3. Causality Assessment 21
Reporting Requirements 21
8.4. 21
8.5. Post-Recruitment Illness 23
9. DATA ANALYSIS/STATISTICAL METHODS 24
9.1. Sample Size Determination 24
9.2. Analysis of Endpoints 24
9.2.1. Analysis of Primary Endpoint assessing Head StART efficacy 24
9.2.2. Analysis of Secondary Endpoints pertaining to Head StART efficacy 25
9.2.3. Analysis of Other Endpoints pertaining to using the RE-AIM framework to evaluate the implementation of the Head StART intervention 25
9.2.4. Analysis of Other Endpoints pertaining to estimating the programmatic cost and budget impact of the Head StART intervention 25
9.3. Safety Analysis 26
10. QUALITY CONTROL (QC) AND QUALITY ASSURANCE 26
11. DATA HANDLING/RECORD RETENTION 27
11.1. Case Report Forms (CRF)/Electronic Data Record 27
11.2. Record Retention 27
11.3. Confidentiality 27
12. ETHICS 28
12.1. Institutional Review Board (IRB) 28
12.2. Ethical Conduct of the Study 28

12.3. Subject Information and Consent 28
13. DEFINITION OF END OF TRIAL..28
14. PUBLICATION OF STUDY RESULTS 29
15. CAPACITY BUILDING...29
16. FUNDING 29
17. REFERENCES 30

1. INTRODUCTION

1.1. Disease Setting/Patient Population

Refugees in sub-Saharan Africa face distinct barriers to Human Immunodeficiency Virus (HIV) care engagement and many individuals newly diagnosed with HIV do not engage in free HIV clinical care. Sub-Saharan Africa is home to nearly 26 million people living with HIV [14], and over 18 million refugees and internally displaced people—a number which has risen dramatically in the last decade [15, 16]. Refugees face unique hardships as they struggle to survive, including difficulty accessing basic needs, disrupted social structures, limited livelihood opportunities, threats to their security, and increased susceptibility to mental health problems [17-22]. Exposure to sexual violence, transactional sex, and poor access to medical services such as treatment and prevention of sexually transmitted infections increase refugees' vulnerability to HIV [17, 19, 22]. Despite free clinical services, including HIV testing and antiretroviral therapy (ART) in refugee settlements across sub-Saharan Africa, many individuals newly diagnosed with HIV in these settings are not engaged in HIV clinical care [2, 21]. To achieve UNAIDS 95-95-95 targets [23], we must identify and implement interventions that facilitate engagement in HIV care for refugees.

Engagement in HIV care for refugees in Uganda may be enhanced by providing more flexible, client-centered HIV services and by optimizing social support. Uganda is the third largest refugee-hosting country in the world, with nearly 1.6 million refugees [4, 24]. Refugee settlements in Uganda are expansive and are located in rural areas. Most refugees that arrive to Uganda leave their country abruptly and travel with only a few relatives, leaving their social networks and possessions behind. Though refugees in Uganda are not restricted legally in terms of movement, most cannot afford to live outside of refugee settlements and rely on subsistence agriculture for survival, given limited livelihood opportunities. In midwestern (MW) and southwestern (SW) Uganda, refugees are predominantly from the Democratic Republic of the Congo (DRC), Burundi, Rwanda, and Somalia, and the main languages spoken include Kiswahili, Kinyarwanda, Runyankore and Somali. In Nakivale Refugee Settlement, a site in SW Uganda with over 150,000 refugees, our refugee-focused research team conducted several studies focused on barriers to HIV care engagement. Of clients newly diagnosed with HIV during routine clinic-based HIV testing from 2013 to 2014, only 54% linked to HIV clinical care in the settlement within 90 days of diagnosis, and 6% initiated ART (PI: O'Laughlin) [2]. Barriers to care included distance from residence to the health center, transport costs, lack of employment, and inclement weather hindering travel [1]. Refugees living with HIV considered community support essential to overcome these obstacles. These findings suggest interventions to provide more flexible HIV services and optimize social support may help overcome barriers to HIV care in this setting [1]. From 2018-2020, our team prospectively followed individuals newly diagnosed with HIV in Nakivale to evaluate factors associated with linkage to HIV care. Of 219 participants newly diagnosed with HIV, 74% linked to HIV care. Presence of social support was the sole factor identified to be significantly associated with linking to care (odds ratio [OR] =2.36, 95% confidence interval [CI]: 1.05-5.34) [3].

1.2. Background and Rationale

Community ART delivery improves HIV outcomes in sub-Saharan Africa. Community ART delivery is a client-centered approach that adapts HIV services to meet the needs of individuals while reducing health system burdens [25]. A community ART delivery intervention for female sex workers in Uganda improved ART adherence from 75% to 95% and viral suppression from 80% to 100% [11]. Community ART groups in Swaziland demonstrated 93.7% 12-month retention on ART [10]. In Mozambique, a study of community ART groups showed high long-term retention in care (98% at 12 months and 92% at 48 months), and a low loss to-follow-up (LTFU) rate of 0.1 per 100 person-years [6]. Also, in Mozambique, community ART groups were associated with 35% lower LTFU [26]. These groups in Mozambique were successful at providing peer support and decreasing barriers to ART, such as travel time to clinic, thereby improving retention in care and health facility capacity [27]. In Lesotho, community ART groups had 99% retention in care at one year compared to 90% for those in standard care as well as improved access to ART [7]. In Zimbabwe, a trial found that retention among community ART groups that were provided multi-month dispensing of ART was similar and non-inferior to that observed in clinic-based care and viral suppression in community ART groups was high with 12-month suppression rates of 99.7% and 92.9% for the quarterly and biannual dispensing groups, respectively [28]. Médecins Sans Frontières noted enhanced retention in care and lightened the burden on the health system with community ART delivery in Malawi, the DRC, South Africa, and Mozambique.[8] Recent studies demonstrate benefit from community ART for clients with unsuppressed viral loads (VLs) and those early in care. The Delivery Optimization of ART randomized trial in Uganda and South Africa included participants not on ART, with a detectable VL, and showed increased viral suppression compared with clinic-based ART (74% vs 63%, relative risk [RR] 1.18) [29]. In South Africa, viral suppression improved after a community ART group intervention for patients with elevated VLs (>400 copies/mL) [30].

Community ART delivery in refugee settlements in Uganda is for stable clients only. Community ART delivery in Uganda is only available for "stable clients" (Uganda Ministry of Health [MoH] [12]) and is offered in two models, client-led and healthcare worker-led groups. Community client-led ART delivery (CCLAD) is a model in which people living with HIV are placed into groups; members rotate collecting ART from the health center for their group and meet at an agreed upon community location to distribute the collected ART. The community drug distribution point (CDDP) model offers similar group-based care in the community, but in this model ART and clinical services (e.g., physical exam, VL monitoring) are brought to the community site by a healthcare worker. Both models provide flexible access to HIV care including ART delivery in the community, as well as psychosocial support provided by group members. Additionally, in both models, clients with HIV are required to receive biannual comprehensive clinical exams and laboratory testing (i.e., VL testing at 6 months, 12 months and then annually). Studies in Uganda indicate high ART adherence for stable clients in CCLAD groups (89%) and CDDP groups (89%) [31], with improvement in ART adherence from 75% to 95% following CCLAD participation among sex workers [11]. Since 2018, CCLAD and CDDP have been offered in refugee settlements in Uganda for stable clients.

The Head StART intervention is grounded in the social ecological model. The social ecological model demonstrates that efforts to modify health behaviors are influenced by levels of constraints [32-36]. To modify behavior, multi-level interventions must take into account these influences on the individual and interactions between levels [32]. In the social ecological model adapted for the refugee settlement context (Figure 1), the levels of influence on health behavior are the *individual*, the *social environment* (the community), the *physical environment* (location and availability of essential resources), and *policies and regulations*. The social ecological model was used by our team to understand barriers to HIV clinic in Nakivale Refugee Settlement in Uganda [1], and has been used in other HIV care continuum research [37-41]. People newly diagnosed with HIV in refugee settlements are at risk of not linking to HIV care unless they are provided accessible care and social support promptly after their diagnosis. The scientific premise of the study is that providing Head StART for clients newly diagnosed with HIV in refugee settlements will positively impact each level of the social-

ecological model. Head StART improves access to ART and lends autonomy (*individual*), decreases community-level stigma (*social environment*), enables participants to travel to clinic less often (*physical environment*), and is an adaptation of existing differentiated ART delivery strategies in place in Uganda (*policies and regulations*).

Summary of significance and gap in existing research. Through its scope (those diagnosed with HIV in the prior 6 months) and target population (refugees), Head StART addresses an important research gap and is likely to improve HIV viral suppression in refugee settlements in Uganda. Providing Head StART promptly after diagnosis is critical as attrition in care often occurs in the first year of ART initiation [42-44], and Head StART can enhance psychosocial support and decrease barriers to ART during this crucial time. Refugees form a population that is particularly vulnerable given disrupted social networks and additional barriers to HIV care in the humanitarian context. By offering refugees Head StART, the mechanisms by which previous community ART delivery programs bolstered retention and viral suppression can help support the transition from linking to care to early viral suppression for this unique population. With large catchment areas and few facility resources to support many people in care, Head StART in refugee settlements presents an opportunity to provide care to individuals who may not be able to engage in traditional models while also reducing the burden on the health system. In the era of COVID-19, reducing health facility encounters through community-based care protects clients and care providers by decreasing risk of SARS-CoV-2 transmission. Findings from the proposed research in Uganda could expand community ART delivery for individuals newly diagnosed with HIV for this community as well as others in sub-Saharan Africa.

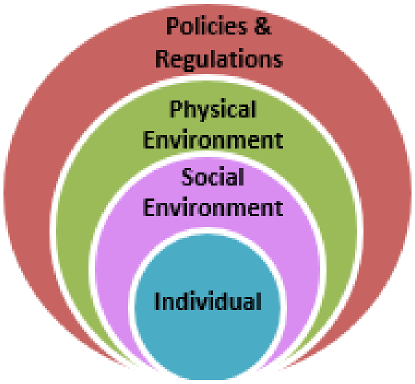

Figure 1. Refugee Settlement Social Ecological Model

2. STUDY OBJECTIVES AND ENDPOINTS

2.1. Objectives

<u>Aim 1</u>: To evaluate the effectiveness of "Head StART," the expansion of community ART delivery to people newly diagnosed with HIV, in achieving HIV viral suppression in refugee settlements in Uganda.
<u>Approach</u>: We will conduct a stratified matched-pair 2-arm cluster randomized controlled trial (RCT) at 12 health centers in refugee settlements in Uganda, in which people diagnosed with HIV within the prior 6 months will be offered community ART delivery. Outcomes for health centers randomized to Head StART will be compared to those at standard care sites.
<u>Primary endpoint</u>: The primary outcome assessed will be the proportion of newly diagnosed subjects with viral suppression at 12 months (+/- 2 months; viral suppression is defined as <1,000 copies/mL).
<u>Secondary endpoint</u>: Secondary outcomes are proportion with viral suppression at 6 months (+/- 2 months), and among virally unsuppressed participants, tenofovir diphosphate (TFV-DP) concentrations in dried blood spots at 12 months (+/- 2 months).
*<u>Hypothesis</u>***:** *Health centers where Head StART is offered will have a higher proportion of clients <u>with viral suppression</u> compared to standard care.*

Aim 2: To assess Head StART implementation across refugee settlement sites to understand the impact of contextual factors on study outcomes.
<u>Approach</u>: Using the RE-AIM implementation science framework, we will evaluate Head StART in terms of its: **1)** <u>Reach</u>: Proportion of eligible individuals at intervention sites participating in Head StART (quantitative) and factors influencing participation (qualitative). **2)** <u>Effectiveness</u>: Perceptions of Head StART benefits and

contributions to ART adherence/ viral suppression (qualitative). **3)** **A**doption: Perspectives of participants and staff on Head StART adoption (qualitative). **4)** **I**mplementation: Adherence to Head StART core components and coverage of intervention (quantitative) and quality of intervention delivery, variation in implementation, and reasons for adaptations (qualitative). **5)** **M**aintenance: Sustained participant effects of Head StART and duration of intervention delivery (quantitative), influence on participants, and reasons for changes over time (qualitative).
Hypothesis: *The Head StART intervention will be most effective in communities with high reach and strong adoption of the intervention.*

Aim 3: To estimate the programmatic cost and budget impact of implementing the Head StART intervention in refugee settlements in Uganda.
Approach: We will conduct activity-based costing and time-in-motion observations to estimate the incremental costs of Head StART implementation. We will combine cost with clinical outcome data from Aim 1 into a Markov model to project the health impact (HIV deaths and morbidity averted) and financial costs of the intervention compared to standard of care (SOC) and to estimate the 5-year budget impact of Head StART implementation.
Hypothesis: *Head StART can be affordably implemented in refugee settlements in Uganda.*

3. STUDY DESIGN

Study design overview. We will randomize health centers (12 health centers total) to intervention sites or standard care and conduct a stratified 2-arm cluster RCT with the primary outcome of proportion with HIV viral suppression at 12 months among newly diagnosed individuals (Aim 1). We will use the RE-AIM framework to assess the Reach, Effectiveness, Adoption, Implementation, and Maintenance of Head StART across study sites (Aim 2). We will estimate the cost and budget impact of the intervention to inform future policy decisions (Aim 3). This trial will help to evaluate the effectiveness of the Head StART intervention on viral suppression while gathering information on implementation (Figure 2).

4. SUBJECT SELECTION

The following eligibility criteria are designed to select subjects for whom protocol treatment is considered appropriate.

4.1. Inclusion Criteria

4.1.1. Inclusion criteria for Head StART intervention participation (intervention participants)

Subjects at intervention sites must meet all the following inclusion criteria to be eligible for participation in the community-based ART delivery intervention:

1. Evidence of a personally signed and dated informed consent document indicating that the subject (or a legal representative) has been informed of all pertinent aspects of the study.

2. Willing and able to comply with scheduled visits, treatment plan, laboratory tests, and other study procedures.

3. Adult ≥18 years of age or mature minors or emancipated minor. Mature minors are defined as individuals 14-17 years of age who have drug or alcohol dependency or a sexually transmitted infection.

Emancipated minors are defined as individuals below the age of majority (18 years) who are pregnant, married, have a child, or are self-sufficient.

4. Tested HIV positive in the past 6 months, not already known to be HIV positive at the time of testing, and not on ART for more than 6 weeks (42 days) at the time of study enrollment.

5. Able to communicate in one of the following 5 study languages: Kiswahili, Runyankore, Kinyarwanda, Somali, or English.

4.1.2. Inclusion criteria for study enrollment (study participants)

To be eligible to be enrolled in the Head StART study and monitored over time through data abstraction, subjects at intervention and standard care sites must meet the following inclusion criteria:

1. Adult ≥18 years of age or mature minors or emancipated minor.

2. Tested HIV positive in the past 6 months, not already known to be HIV positive at the time of testing, and not on ART for more than 6 weeks (42 days) at the time of study enrollment.

4.2. Exclusion Criteria

4.2.1. Exclusion criteria for study enrollment and Head StART intervention participation

Subjects presenting with any of the following at initial screening will not be enrolled in the Head StART study and will not be offered participation in the Head StART intervention:

1. Pregnant and breastfeeding women.
2. Clients deemed by the clinician(s) unfit for community-based care secondary to medical need.
3. Concurrently enrolled in another biomedical clinical trial.

4.3. Waiver of consent

We will seek a waiver of consent to follow the cohort of newly diagnosed individuals and collect the relevant clinical outcomes for all individuals that meet eligibility requirements at intervention and standard care sites. A waiver of consent is necessary because it is not practicable to obtain consent from these individual before beginning the study as we will not be in contact with them until they come for 12-month viral load testing. Additionally, some individuals may not return for 12-month viral load testing and we need to be able to collect clinical care data about them for the validity of the study.

4.4. Randomization Criteria

To conduct this cluster RCT, we identified 12 health centers. Health center sites were stratified based on 1) region in the country, 2) refugee settlement, 3) catchment population demographics, 4) clinic volume, and 5) number of clients newly diagnosed with HIV in the preceding year (Table 1, next page). Grouping based on these characteristics yield the following 4-2-2-4 stratified matching approach:

Group A) SW/ Nakivale Refugee Settlement (N=4): Nakivale, Juru, Kibengo, Rubondo

Group B) SW/ Oruchinga Refugee Settlement (N=2): Rulongo, Nshugyezi

Group C) SW/ Rwamwanja & Kyaka Refugee Settlements (N=2): Rwamwanja, Bujubuli

Group D) MW/ Kyangwali Refugee Settlement (N=4): Kasonga, Maratatu, Kyangwali, Rwenyawawa

Table 1. Head StART health center randomization strata

Health Center	Region	Refugee Settlement	Consults/Month*
Nakivale HC III	SW	Nakivale	8,120
Kibengo HC III	SW	Nakivale	2,449
Rubondo HC II	SW	Nakivale	4,659
Juru HC III	SW	Nakivale	3,325
Rulongo HC III	SW	Oruchinga	1,625
Nshungyenzi HC III	SW	Oruchinga	2,407
Rwamwanja HC IV	MW	Rwamwanja	5,350
Bujubuli HC IV	MW	Kyaka	3,630
Kasonga HC II	MW	Kyangwali	1,200
Maratatu D HC III	MW	Kyangwali	3,100
Kyangwali HC IV	MW	Kyangwali	2,500
Rwenyawawa HC III	MW	Kyangwali	1,600

*Consults= outpatient department consults; SW= southwest, MW= Midwest

Within each group, we will randomize half to the intervention and half to standard care. Randomization will be performed publicly at the Head StART study inception meeting prior to research implementation. For the randomization, four bowls will be prepared, one for each randomization group, contain identical colored balls each with a slip of paper with the name of one of the health center sites inside of it. Four stakeholders will be invited to randomly select half the balls in each bowl (i.e., two balls for Groups A & D and one ball for Groups B & C. The health center sites in the selected balls will become intervention sites and the health center sites in the remaining balls will become standard care sites. Randomization will occur prior to research implementation. Eligible individuals at intervention sites will be offered the Head StART intervention and eligible individuals at both intervention and control sites will be followed prospectively for study outcomes. Intervention participants must consent to participate in the intervention and will have the option to instead receive standard care (facility-based HIV care). Though participation is optional, analysis will occur at the level of the health center with an intention-to-treat analysis to help overcome participation bias.

4.5. *Other*

For intervention participants identified during the study period as pregnant and/or breastfeeding, community ART delivery will be discontinued and they will be referred to the antenatal clinic and/or the prevention of mother-to-child transmission (PMTCT) of HIV clinic. When deemed necessary by clinic staff, intervention participants with advanced disease or significant comorbidities will be shifted from community ART delivery to facility-based care. Intervention participants referred back to facility-based care may return to the Head StART intervention (i.e., community-based ART delivery) when their health concerns resolve.

5. TREATMENTS OF SUBJECTS

5.1. Allocation to Treatment/Group

Head StART intervention. As described in section 4.4 above, within each stratum, health centers will be randomly selected as a Head StART intervention site or a standard care site. At intervention sites, newly enrolled individuals who consent to participate in the intervention will be assigned to a community ART delivery group. The community ART delivery group will either be a CCLAD group or a CDDP group based on participant preference as well as proximity of an existing group to the enrollees' home residence. Given the reality that stable clients may move between CCLAD and CDDP groups, it will not be feasible to separate these community ART delivery models for the purposes of this study, though we will collect data on which group participants are engaged in for which length of time for secondary analyses. Newly diagnosed individuals (diagnosed in the prior 6 months) at intervention sites will be offered community ART delivery on a rolling basis during the 3-year enrollment window, initiating individuals into groups as close to their date of diagnosis as possible. Newly enrolled individuals will join existing community ART delivery groups, or when a new group is needed based on group size or geographic location, a new group will be formed. Intervention participants will not be compensated for attending community ART delivery group meetings. The HIV health worker will explain group member roles and responsibilities (as guided by the Ugandan MoH "Implementation Guide for Differentiated HIV Services in Uganda"[12]). Intervention participants will be advised about the importance of maintaining confidentiality as some group members may not want to disclose their HIV status to others in the community. At sites randomized to standard care, study participants newly diagnosed with HIV will receive facility-based care per Uganda MoH protocols [13] (Table 2). The trial is not blinded, health center staff and participants will know if they are located at an intervention site. The investigator's knowledge of the treatment should not influence the decision to enroll a particular subject or affect the order in which subjects are enrolled. There is low likelihood of contamination between study sites given they are geographically dispersed, and many participants have limited access to transportation making travel to other locations difficult.

5.2. Breaking the Blind

Not applicable

5.3. Drug Supplies

Not applicable

5.3.1. Administration

Not applicable

5.3.2. Drug Storage and Drug Accountability

Not applicable

5.4. Concomitant Medication(s)

Not applicable

Table 2. Standard care compared to Head StART intervention

	Standard care	Head StART intervention	
		**CCLAD**	**CDDP**
**Leadership**	Health worker	Client	Health worker & CASAs*
**Meeting location**	Clinic	Community	
**Frequency**	≥4x/year	≥4x/year	
**ART pick up (every 3 months)**	At clinic	(Rotating) client at clinic	Delivered to community
**Biannual clinical check up**	At clinic	At clinic	In community
**VL* check (6, 12 months)**	At clinic	At clinic	In community
**Psychosocial support provision**	At clinic	Group members, health worker	Group members, health worker, CASAs*

*CCLAD= community client-led ART delivery groups, CDDP= community drug distribution point groups, CASA= Community ART support agents, VL=viral load

6. STUDY PROCEDURES

6.1. Screening

At intervention and non-intervention sites, individuals will be screened for possible inclusion in the study by research staff who will review HIV testing registers, HIV clinic records, pharmacy dispensing records and by active referral by HIV Counsellors and other clinic staff. Potential subjects will be approached in-person as well as over the phone when possible. Given the intention to treat analysis plan, we have a waiver of consent to follow the eligible cohort prospectively and collect the relevant clinical outcomes for all individuals that meet eligibility requirements at intervention and standard care sites.

6.2. Study Period

This study will take place over five years (Table 3). In year one, the first six months will be dedicated to finalizing ethics and local/national approvals, recruiting, and hiring research staff, and training research staff. We will start participant recruitment and enrollment once necessary study approvals are granted at ~ six months and will continue for three years until ~ 3.5 years into the five-year study period. We will continue Head StART groups until the end of the 5-year study period to maximize data collected on retention outcomes.

Table 3. Protocol activities and approximate study timeline

Protocol activity	Years				
	1	2	3	4	5
**Study implementation preparation**					
**Aim 1: Head StART intervention effectiveness**					
Identification of cohort at intervention and control sites and enrollment/informed consent at intervention sites					
Data collection follow-up period at all sites (clinic/group attendance, ART pick up, viral loads at 6- and 12- months, TFV-DP levels at 12 months)					
**Aim 2: Assess Head StART implementation using RE-AIM**					
Data collection					
Analysis					
**Aim 3: Estimate program costs and budget impact analysis of Head StART**					
Data collection					
Analysis					
**Dissemination of results**					
Dissemination of study progress and findings to stakeholders		X X	X X	X X	X X

Throughout the study, project updates will be disseminated to stakeholders in Uganda approximately twice each year. Presentations will be made at national and international conferences. In addition, final results will be disseminated to stakeholders in Uganda and sub-Saharan Africa at the conclusion of the study.

6.3. Follow-up Visit

Clinic attendance. Individuals may attend clinic at the facility (standard of care and intervention sites) or in the community (intervention sites). These clinic attendance data will be abstracted monthly by research assistants (RAs) at both sites. For intervention participants, biannual comprehensive clinical evaluations will either occur at the HIV clinic (CCLAD model) or in the community (CDDP model) (Table 2). Intervention participants can attend the clinic at any time, including when they are unwell, have questions for the health worker, if laboratory testing is needed, and if requested by the health worker.

Viral load (VL) testing will take place per Ugandan Ministry of Health (MoH) guidelines. Blood for VL samples will be drawn by laboratory personnel either at the clinic (standard care group, CCLAD model) or in the community (CDDP model) per Ugandan MoH guidelines (Table 2).

Tenofovir diphosphate (TFV-DP) dried blood spot sample collection. During standard VL testing at ~12 months from HIV diagnosis at intervention and standard care sites, both individuals enrolled in study procedures (intervention participants) and individuals being followed in the standard care cohort, will be invited to give their consent to have some of the whole blood that was drawn for VL testing stored for potential TFV-DP concentration testing. This will require a small portion (25µL) of the whole blood sample taken by a single blood draw as part of standard VL testing to be placed on a dried blood spot card before the sample is spun down for plasma. A random sample of individuals identified to have unsuppressed VLs will have their TFV-DP concentrations measured using their dried blood spot sample to evaluate ART adherence as a possible mechanism for being virally unsuppressed. We will attempt to sample all those unsuppressed, but if limited by budget constraints will perform TFV-DP testing for a random sample of unsuppressed participants in each group. As TFV-DP testing is not a part of routine laboratory testing, participants will provide written consent to have this laboratory test and will be remunerated with 20,000 UGX (~$5.67 USD) to cover the cost of transportation and their time. They will also provide consent for blood sample storage.

6.4. Subject Withdrawal

Stopping Head StART intervention. Subjects may withdraw from the Head StART intervention (i.e., community-based ART delivery) at any time at their own request, or they may be withdrawn at any time at the discretion of the investigator or sponsor for safety or behavioral reasons, or the inability of the subject to comply with the protocol required schedule of study visits or procedures. Finally, intervention participants (i.e., receiving community-based ART) may be referred to facility-based care by health care staff at any time during the study period for health concerns (e.g., newly pregnant or breastfeeding women, newly deemed by a clinician to be unfit for community-based care secondary to medical need). Individuals changed from the community-based care intervention to facility-based care will be asked to participate in a brief change in care model/exit interview to facilitate understanding of what aspects of the intervention were acceptable or not acceptable to them. Study data will continue to be abstracted for these individuals. Intervention participants referred back to facility-based care may return to the Head StART intervention (i.e., community-based ART delivery) when their health concerns resolve.

Intensive Outreach Team to minimize loss-to-follow-up (LTFU) and missing data. Intensive Outreach Teams will be in place at each health center to optimize end of study assessments of the 12-month VL and TFV-DP concentration level testing for those with unsuppressed VLs. These teams will work to notify and encourage those who did not return for their 12 months testing to complete this assessment. Intensive Outreach Teams will facilitate communication outreach, movement in the community, and additional operational expenses. To ensure Intensive Outreach Team activities do not alter the intervention, their efforts will begin at the 12-month point (i.e., time of primary outcome data point) and not the 6-month VL testing point (secondary outcome). If a subject does not return for a scheduled visit, every effort should be made to contact the subject. In any

circumstance, every effort should be made to document subject outcomes, if possible. The investigator should inquire about the reason for withdrawal and follow-up with the subject regarding any unresolved adverse events.

Termination from study. Study participants will reach the end of their study involvement if they die or if it is the end of the study period. In all instances of death, investigators will complete a case report form (CRF).

7. ASSESSMENTS

7.1. Efficacy

Data variable collection and management. Data will be collected approximately monthly at intervention and control sites by data clerks in place at the health implementing partner clinics. Data will be abstracted from written HIV clinic, laboratory registers, pharmacy registers, as well as abstracted from MoH community ART delivery registers (includes CCLAD and CDDP groups), and entered directly into passcode protected, encrypted, electronic devices. Data will be uploaded by the data clerks to a secure web-based platform with off-line capacity. Variables to be collected include ART initiation/start date, ART regimen, ART medication pick-up, VLs/date of test, and HIV clinic/community ART group visit dates. These data elements will be collected for all eligible individuals at intervention and control sites, regardless of participation in the intervention for those at intervention sites, with an intention-to-treat analysis.

7.2. Implementation assessment

RE-AIM Implementation Science Framework to assess implementation. We will use a mixed-methods approach guided by the RE-AIM framework to assess the Reach, Effectiveness, Adoption, Implementation and Maintenance [45-47]. To do this, the quantitative and qualitative measures demonstrated in Table 4 below will be assessed. Quantitative data will be collected by data clerks at each health center during the enrollment period, and abstracted from HIV clinic, laboratory, and pharmacy registers as well as from MoH community ART group registers. Data will be collated prospectively. Qualitative data will be obtained via in-depth interviews for all RE-AIM domains, with direct observations for assessing Adoption, Implementation, and Maintenance. We will purposively select participants to ensure we include refugees from different countries of origin as well as Ugandan nationals. We anticipate we will conduct no more than 40 interviews and we will be prepared to stop earlier if saturation is reached. We will include interviews around 0, 6, and 12 months with intervention participants who are at intervention sites (~ 20), as well as with intervention eligible non-participants at intervention sites (~10), with health center staff/leaders and with policy leaders (~10). We will interview intervention participants and eligible non-participants at intervention sites over time to better understand the influence of Head StART on HIV care engagement as well as differences over time.

Interview participants will be assured that their responses will be kept confidential and that their decision to participate in the interviews will not affect access to or quality of their HIV care services. Interview participants will be reminded that they are not be required to take part and that their responses will not be linked to their names. All interviews will be scheduled at a time and location convenient for the participant with the goal of

Table 4. RE-AIM measures to assess the Head StART intervention compared to standard care

Dimension	Quantitative Measures	Qualitative Measures*
**Reach:**<br>Proportion that participates	- Proportion of eligible individuals participating in the intervention at intervention sites	- Explore factors influencing Head StART participation (e.g., stigma, social support, distance/cost to clinic)
**Effectiveness:**<br>Impact of Head StART on outcomes	- Viral suppression (<1,000 copies/mL) at 12 months (Aim 1)	- Understand benefits and any negative effects of Head StART (e.g., peer support, access to ART, stigma)<br>- Learn about influences of Head StART on ART adherence
**Adoption:**<br>Head StART components embraced	*Adoption of Head StART by setting and staff is fixed by the study design*	- Perspectives of participants/health staff on Head StART (e.g., relative advantage, compatibility, complexity, cost, risk)
**Implementation:**<br>Fidelity and consistency of use	- Adherence to Head StART components (% adherence to core components)[12,89]<br>- Coverage of Head StART (% offered Head StART)	- Explore quality of intervention delivery (direct observation) and interactions with participants[89]<br>- Understand reasons for staff practice variations/adaptations in implementation
**Maintenance:**<br>Consistency over time and settings	- Individual level: extent Head StART effects are sustained (i.e., VL and ART drug levels)<br>- Health center level: duration of high-fidelity Head StART delivery over time and after intervention period	- Understand influence of Head StART over time on individuals participating in the intervention<br>- Learn from health center staff/leaders and policy leaders about reasons for differences in Head StART comparing years 1-5 of the study period

*Participants in Head StART intervention (N=20, interviews at the following time points: 0, 6, and 12 months), Non-participants at Head StART health center sites (i.e., opted out) (N=10), health policy leaders, clinic leaders and staff (N=10); total N=40.

maximizing confidentiality. Multilingual RAs trained in qualitative techniques will conduct private interviews less than one hour in length in the participant's preferred language. Interviews will be audio-recorded with permission and transcribed verbatim by the RA. Interviews will follow a semi-structured interview guide. We will provide 20,000 Ugandan shillings (~5.28 USD) at the qualitative interview as reimbursement for participant's time and transportation costs.

Direct observation by research team members will occur monthly at each health center on randomly selected days. RAs will use structured checklists to evaluate the Head StART components delivered to the clients. In addition, observers will provide detailed, open form notes on Head StART delivery. These observations will help facilitate Head StART evaluation across implementation sites (i.e., adoption, implementation, maintenance).

7.3. Programmatic cost and budget impact assessment

We will conduct micro-costing, staff interviews, and time and motion observation to estimate the cost of implementing Head StART as part of routine HIV care in refugee settlements. Costs of the intervention and standard care activities will be collected using the payer (Ugandan MoH) perspective. A trained RA will utilize standardized Excel cost menus to collect intervention costs: including start-up (e.g., HIV clinic worker training), human resources, supplies, participant transport reimbursement. We will conduct interviews with HIV clinic workers to assess daily responsibilities associated with implementing Head StART (Appendix C). We will conduct time-motion observations in a representative subset of HIV clinics to assess variation in Head StART implementation by setting (e.g., clinic volume, clinic population). We will obtain permission from participants to observe patient/provider interactions. We will utilize published estimates of ART drug costs, laboratory monitoring, and HIV-related hospitalizations to estimate program costs incurred and averted by Head StART implementation. Research time (e.g., informed consent) will be removed from programmatic costs.

7.4. Pharmacokinetics Assessments

7.4.1. Dried blood spot samples for analysis of Tenofovir diphosphate (TFV-DP)

Dried blood spot (DBS) samples will be prepared from the whole blood samples collected for VL testing for quantification of tenofovir diphosphate (TFV-DP) concentrations at approximately 12 months from subjects' initial HIV diagnosis. TFV-DP concentrations will be quantified using a validated assay.

7.4.2. Justification for PK/other sample shipment

The DSB samples for quantification of intracellular TFV-DP concentrations will be shipped on dry ice to an accredited and certified laboratory with the ability to conduct this processing. Sample transfer and analysis will be governed by a material transfer agreement which will be signed between both parties. We will seek permission for participants for storage and future use of samples.

7.5. Pregnancy Testing

We will not be conducting pregnancy testing as a part of this study. We will rely on subject self-report of pregnancy test results and/or pregnancy testing conducted as a part of routine care at the health center to help identify pregnant women that will be excluded from participating in the study.

8. ADVERSE EVENT REPORTING

8.1. Terminology

8.1.1. Non-Medical Adverse Event (Non-Medical AE)

An adverse non-medical event is any untoward or unfavorable occurrence in a human subject that is not a medical occurrence. The research may or may not be causally related to it.

8.1.2. Medical Adverse Event (Medical AE)

An adverse event is defined as any untoward or unfavorable *medical* occurrence in a human subject, including any abnormal sign (for example, abnormal physical exam or laboratory finding), symptom, or disease temporally associated with the subject's participation in the research. The research may or may not be causally related to it.

8.1.3. Non-Medical Serious Adverse Event (Non-Medical SAE)

A serious non-medical event is a serious untoward or unfovorable occurrence in a human subject that is not a medical occurrence. The research may or may not be causally related to it.

8.1.4. Medical Serious Adverse Event (Medical SAE)

A serious medical event is defined as an adverse medical event (see above) that is life threatening, results in death, hospitalization, disability or permanent damage, congenital anomaly/birth defect, or other serious important medical event. The research may or may not be causally related to it.

8.2. Severity Assessment

The severity of AEs will be graded according to the Division of AIDS (DAIDS) Table for Grading the Severity of Adult and Pediatric Adverse Events [51].

8.3. Causality Assessment

All (S)AEs will be assessed for causality (e.g., by exploring temporal relationships, alternative explanations, and assessing frequency of occurrence outside the research context) to discern if there is a reasonable possibility that study participation caused or contributed to the (S)AE. The relationship of the study procedures (i.e., early community-based ART delivery) to each (S)AE will be assessed using the following definitions:

RELATED: There is a reasonable possibility that the (S)AE may be related to the study.

NOT RELATED: There is not a reasonable possibility that the (S)AE is related to the study.

8.4. Reporting Requirements

Non-medical AEs: *Anticipated* non-medical AEs that are deemed unrelated to study activities (per the judgement of the PI and Site PI) will not be recorded or reported. Examples include the following:

- Routine domestic quarrels
- Inadvertent disclosure of HIV status
- Perceived or experienced stigma
- Mild/moderate psychological symptoms (e.g., anxiety, stress)

However, if these non-medical events are *unanticipated* and are deemed to be related to study activities, they will be recorded and reported to the DMC in the biannual report and to the IRB in the annual report.

If these non-medical events result in major consequences per the judgement of the PI and Site PI (e.g., dissolution of a relationship, necessity to move residence, and/or hospitalization), they will be considered recorded as non-medical SAEs and reported within 7 days.

As study participants will be those newly diagnosed with HIV, the following are some of the *anticipated* medical AEs that will not be recorded or reported to ethics review boards as they are not directly related to study-specific activities:

- Side effects from anti-retroviral therapy (ART) including Immune Reconstitution Inflammatory Syndrome (IRIS)
- Mild/moderate symptoms and illnesses related to HIV
- Mild illnesses from common infections (e.g., malaria, pneumonia)

Further, as study participants are living in or near a refugee settlement and as such are likely to face dangers in day-to-day life and common ailments that are not related to study participation, the following are examples of anticipated medical SAEs:

- Injuries or death resulting from road traffic accidents (including motorcycles, cars and trucks)
- Severe illnesses from common infections (e.g., malaria, pneumonia)
- Complications related to childbirth

Should an *anticipated* medical SAE occur, it will be reported to the DMC in the biannual report and to the IRB in the annual report.

Given the known safety of ART medications for treatment, and the focus of this study on early community ART delivery rather than the medications themselves, we will not report the *anticipated* adverse non-medical and medical events as described above. We will only report *unanticipated* non-medical/medical AEs and any non-medical/medical SAEs on study case report forms. All non-medical/medical SAEs will be reported regardless of whether they are *anticipated* or *unanticipated.* The definition of an *unanticipated event* for this purpose of this study includes *an event that meets all of the following criteria: a) unexpected, b) reasonable probability that the event is related to study, and c) places subjects at greater risk of harm*. If an *unanticipated* non-medical/medical AEs and any non-medical/medical SAE occurs, reporting will follow institutional review board (IRB), local and international regulations as appropriate (event reporting timeline outlined in Table 5).

For all *unanticipated* non-medical/medical AEs and any non-medical/medical SAEs, the investigator must pursue and obtain information adequate to determine the outcome of the event and to assess whether it meets the criteria for classification requiring urgent reporting (Table 5). Additionally, sufficient information should be obtained by the investigator to determine the causality of the event. Follow-up by the investigator may be required until the event or its sequelae resolve, return to baseline, or stabilize at a level acceptable to the investigator.

Table 5. Schedule for safety and protocol deviation reporting

Type of Event	Anticipated vs Unanticipated	Report to UW Principal Investigator	Report to Data Monitoring Committee (DMC)	Report to Makerere IDI-REC IRB
non-medical AE	Anticipated	n/a	n/a	n/a
	Unanticipated*	Within 24 hours	In the biannual meeting and report	Within 7 business days
medical AE	Anticipated	n/a	n/a	n/a
	Unanticipated*	Within 24 hours	In the biannual meeting and report	Within 7 business days
non-medical SAE	n/a	Within 24 hours	In the biannual meeting and report	Within 7 business days
medical SAE	Anticipated	Within 24 hours	In the biannual meeting and report	In the annual report
	Unanticipated*			Within 7 business days
Protocol deviation (minor error, no harm/change)	n/a	Within 7 days	In the biannual meeting and report	In the annual report
Protocol violation	n/a	Within 24 hours	In biannual committee meeting	Within 7 business days

*For events also deemed to be related to the study
AE= adverse event, SAE=serious adverse event

Any other unanticipated problems, compliance issues, or safety concerns identified by the study team will be evaluated by the study team to assess severity, causality, and the appropriate next steps.

8.5. Post-Recruitment Illness

As this study involves assessment of an ART delivery strategy and is not evaluating the efficacy of a study drug, the study team will monitor post-recruitment illness outcomes when the illness is deemed to be an *unanticipated* medical AE or SAE related to study participation. However, provision of medical care will be provided by the health implementing partner and the Ugandan Ministry of Health per standard protocols and will not be provided by the Head StART study.

9. DATA ANALYSIS/STATISTICAL METHODS

9.1. Sample Size Determination

Viral suppression for individuals accessing services in refugee settlements in sub-Saharan Africa is notably low [52]. We believe that participants newly diagnosed with HIV in the Head StART intervention will experience an additional 20%-point increase in viral suppression at 12 months (primary outcome) compared to those not participating in Head StART. To account for less than perfect participation in the intervention at the intervention sites (we conservatively estimate 75% participation based on willingness to participate in the pilot data; in the pilot, the overall proportion willing to participate was 80% and the site with the lowest proportion willing was 75%), we assume an average effective size of 15% points (20% point * 0.75 participation= 15% points) in viral suppression at the intervention sites. Overall, we anticipate 60% viral suppression [<1000 copies/mL at 12 months] at control sites compared to 75% viral suppression at intervention sites. An RCT with a behavioral intervention assessing HIV outcomes in sub-Saharan Africa was powered for comparable effective sizes using an intention-to-treat analysis [53], and an observational study in Uganda and South Africa demonstrated a 15% improvement in viral suppression after a home-based HIV care intervention [54]. Assuming an inter-cluster correlation of 0.02, power of 80%, 5% margin of error (2-sided), the required sample size is 1,360 newly diagnosed per arm (total 2,720) using the 4-2-2-4 stratified randomization approach. To account for additional loss to follow up, we will aim to enroll up to 1,560 per arm (up to 3,120 total). Based on the number of clients newly diagnosed with HIV in the study site health centers from July 2021-June 2022, we are confident we will reach our sample size targets (Table 6, projected enrollment over 3 years: 3,408).

Table 6. Projected # participants contributing to outcome

Health Centers	New HIV+ adults* (7/2021-6/2022)	Projected # participants
Nakivale HC III	159	477
Kibengo HC III	58	174
Rubondo HC II	50	150
Juru HC III	41	123
Rulongo HC III	47	141
Nshungyenzi HC III	112	336
Rwamwanja HC IV	174	522
Bujubuli HC IV	174	522
Kasonga HC II	50	150
Maratatu D HC III	61	183
Kyangwali HC IV	152	456
Rwenyawawa HC III	58	174
TOTAL (3-years of enrollment): (1,704/arm)		3,408

*Pregnant/breastfeeding women and individuals < 18 years old excluded

9.2. Analysis of Endpoints

9.2.1. Analysis of Primary Endpoint assessing Head StART efficacy

We will conduct an intention-to-treat analysis of the primary outcome (viral suppression defined as <1000 copies/mL, consistent with Uganda MoH definitions[13, 55]) at 12 months. Our primary analysis will estimate the difference in the prevalence of viral suppression comparing the intervention and standard care clusters using a two-stage approach recommended for cluster-randomized trials with a small number of clusters per group [56]. We will calculate prevalence ratios comparing the proportion of those with viral suppression in the two study arms by taking the geometric mean of the prevalence ratio observed in each of the matched site pairs and constructing a 95% CI using a normal approximation [56]. Descriptive summaries of the quantitative measures will be presented for the intervention and control groups. Descriptive statistics will be used to compare categorical and continuous variables. If primary outcome data are missing or in cases of LTFU, we will assume individuals are not virally suppressed to fit the most conservative models. We will assess patterns to missingness and impute missing data (e.g., multiple imputation or sensitivity analysis using assumptions about participants with missing primary outcomes). Prior research in sub-Saharan Africa demonstrated that 71% of participants lost to follow-up had high viremia (>1000 copies/mL) [57]; we will therefore assume the prevalence of viral suppression to be ~30% among those without VL test results at the specified time points.

9.2.2. Analysis of Secondary Endpoints pertaining to Head StART efficacy

Secondary outcomes include viral suppression at 6 months and more stringent viral suppression thresholds used in recent trials (<400 copies/mL and <200 copies/mL) [58-61]. We will assess TFV-DP concentration mean, median and IQRs for each study arm and will use linear regression to evaluate potential differences in values between the intervention and control sites. Using a 3-level categorization for TFV-DP levels, we will describe the proportions of low, medium, and high adherence for each study arm and compare levels by intervention group using ordinal logistic regression models, clustering by site.

9.2.3. Analysis of Other Endpoints pertaining to using the RE-AIM framework to evaluate the implementation of the Head StART intervention

Quantitative considerations. We will conduct a secondary stratified analysis by health center to assess the potential for outcome differences by location (Aim 2). Descriptive summaries of the quantitative measures will be presented by health center (Table 4). Log-linear regression will be used to compare differences in the proportion of eligible individuals participating in the intervention between sites. Bivariate analysis will be used to explore the association between the additional quantitative outcome measures outlined in Table 4 and key demographic variables such as age, sex, and refugee/national status.

Qualitative analysis. To analyze the qualitative data, two coders will read a randomly selected interview to identify general themes using the RE-AIM domains to initially organize the data. A content analysis approach will then be used to identify additional themes under each RE-AIM domain to demonstrate determinants influencing Reach, Effectiveness, Adoption, Implementation, and Maintenance of the Head StART intervention [62]. The coders, with the help of the qualitative research team, will create a preliminary codebook, discuss initial findings, and resolve discrepancies. The codebook will be developed iteratively by coding an additional subset of transcripts together. Investigators will use the revised codebook to code the remaining interviews. For analysis of the direct observation data to understand Head StART adoption, implementation, and maintenance at the health center sites, the coding process will be iterative as we expect changes in adoption to occur over time. At the conclusion of the study, key themes will be described, addressing potential variability observed over time and differences by study site.

Integration of quantitative and qualitative data. Through convergence and complementarity, we will connect quantitative and qualitative results, using qualitative data to provide depth of understanding [63-65]. The concurrent collection of quantitative and qualitative data design provides an opportunity for the quantitative results to inform subsequent qualitative data collection, which can be useful if the quantitative results are unexpected [66]. Similarly, we will use quantitative data to evaluate assertions arising from the qualitative data.

9.2.4. Analysis of Other Endpoints pertaining to estimating the programmatic cost and budget impact of the Head StART intervention

We will combine costs and outcome data from Aim 1 into an Excel-based Markov model parameterized to reflect HIV natural history and care engagement of refugees in Uganda. We will use the model to project the annual costs and budget impact of implementing Head StART, incorporating the eligible refugee population size, current and future intervention mix (SOC and Head StART), and total health expenditures. We will examine HIV treatment budgets, HIV prevention, refugee health budgets, and personnel budget allowances. Following guidelines established by The Professional Society for Health Economics and Outcomes Research [67], we will utilize a 5-year time horizon. We will model two scenarios 1) Head START implementation and 2) standard care. We will estimate the HIV-related deaths and morbidity averted in Head StART versus standard care. We will assess the budget impact of Head StART accounting for costs incurred and averted by the intervention (e.g., incremental costs of standard care ART refill visits compared to costs averted by reduced clinic visits with Head StART, hospitalizations averted due to higher ART adherence in the Head StART

scenario) as the difference in cost between the intervention and standard care scenario. We will conduct univariate and probabilistic sensitivity analyses to assess the influence of model assumptions. We will conduct Monte Carlo simulation to generate uncertainty intervals around cost estimates. We will also assess variations in budget impact by HIV clinic size, location, and number of clients newly diagnosed with HIV. Secondary model outcomes will include viral suppression and HIV deaths averted in the Head StART scenario compared to SOC over the 5-year time horizon.

9.3. Safety Analysis

We will compare unanticipated adverse event rates and serious adverse event rates between the two study arms (i.e., the intervention and standard care arms).

9.4 Interim Analysis

Not applicable

9.5 Data Monitoring Committee (DMC)

The Head StART DMC will consist of a minimum of three independent external reviewers, including a biostatistician, a clinician with expertise in HIV, and a researcher with expertise conducting clinical trials and implementation research in sub-Saharan Africa. Dr. O'Laughlin (PI) and Dr. Muwonge (Site-PI) will select DMC members with appropriate expertise and without conflicts of interest. Prior to study start, the DMC will meet to review the protocol and ensure all necessary information is available to evaluate the study; the goal of the DMC will be to monitor study implementation, data quality, outcomes, and *unanticipated* non-medical and medical AEs and *all (anticipated and unanticipated)* non-medical and medical SAEs. Prior to study implementation the DMC will define voting rules. The DMC will periodically review and evaluate the accumulated data for participant safety, study conduct and progress, and make recommendations to the study investigators regarding the need for any modification or termination of the trial. The DMC will meet every six months by video-conference call; the cadence of the meetings will be readdressed during each meeting and the frequency adjusted as needed. In addition, the DMC members and the PI will be in regular email and phone contact regarding the progression of the study protocol and the response to *unanticipated* non-medical/medical AEs and all non-medical/medical SAEs.

The study team will prepare reports for the DMC twice a year, which will include summary reports of patient safety data and protocol deviations. The DMC Chair has the responsibility of calling an ad hoc meeting if the type or frequency of *unanticipated* non-medical/medical AE or any non-medical/medical SAE is of concern. The DMC may request additional information from the PI at any time during its review of the study. The DMC will ensure documentation of informed consent and review confidentiality procedures. Each year, a DMC report will be produced that summarizes: 1) *unanticipated* non-medical/medical AEs and *all* non-medical/medical SAEs, 2) the committee's opinion as to whether safety, confidentiality, and privacy have been assured by the investigators, and 3) progress towards recruitment and follow-up goals. The yearly DMC summary will be forwarded to the PI. The PI will then submit the summary to the Makerere University Infectious Diseases Institute Research and Ethics Committee (IDI-REC), the University of Washington Human Subjects Division, and to the National Institute of Mental Health (NIMH).

10. QUALITY CONTROL (QC) AND QUALITY ASSURANCE

Data monitoring and quality assurance will be done on an ongoing basis. Dr. O'Laughlin (PI) and/or Dr. Muwonge (Site-PI) will monitor the study operations and facilitate prompt reporting of medical and non-

medical unanticipated and serious AEs and other study-related information in study documents and to the IRB as appropriate. Team meetings by the PI, Site-PI, and study staff will be held approximately twice monthly to discuss protocol issues and to review AEs. All data will be collected/abstracted and stored on passcode protected, encrypted, electronic devices. The Project Coordinator will monitor study data every month to safeguard the accuracy and completeness of consent forms and other study documentation. To ensure patient safety, data and safety monitoring will include review of all reportable AEs, enrollment, and protocol deviations, in addition to review of any interim analyses to ensure confidentiality is maintained. Field staff will be responsible for reporting medical and non-medical unanticipated AEs and serious AEs and breach of patient confidentiality, to the Project Manager and to the PI (Dr. O'Laughlin) and the Site-PI (Dr. Muwonge). The PI or the Site-PI will notify the Makerere University IDI-REC, UNCST, and the University of Washington Human Subjects Division, of any breaches in confidentiality or reportable AEs and events attributable to this study according to Table 5. Senior oversight for data safety and monitoring will be provided by the Research Internal Monitor based at the Infectious Diseases Institute at Makerere University.

During study conduct periodic monitoring may be conducted to ensure that the protocol and Good Clinical Practices (GCPs) are being followed. The monitors may review source documents to confirm that the data recorded on CRFs is accurate. Additionally, the study site may be subject to review by the IRB and/or to inspection by appropriate regulatory authorities.

11. DATA HANDLING/RECORD RETENTION

11.1. Case Report Forms (CRF)/Electronic Data Record

A CRF is required and should be completed for each included subject.

The investigator has ultimate responsibility for the collection and reporting of all clinical, safety and laboratory data entered on the CRFs and any other data collection forms (source documents) and ensuring that they are accurate, authentic / original, attributable, complete, consistent, legible, timely (contemporaneous), enduring and available when required. The CRFs must be signed by the investigator or by an authorized staff member to attest that the data contained on the CRFs is true. Any corrections to entries made in the CRFs, source documents must be dated, initialed and explained (if necessary) and should not obscure the original entry.

11.2. Record Retention

To enable evaluations and/or audits, the investigator agrees to keep records, including the identity of all participating patients (sufficient information to link records, e.g., CRFs and hospital records), all original signed informed consent documents, copies of all CRFs, safety reporting forms, source documents, and detailed records of treatment disposition, and adequate documentation of relevant correspondence (e.g., letters, meeting minutes, telephone calls reports).

Investigator records must be kept for as long as required by applicable local regulations (UNCST generally requires a minimum of 5 years). When more than one requirement can be applied, records must be maintained for the longest period provided.

11.3. Confidentiality

Clinical data will be entered into a study specific database by designated staff on a regular basis from completed Case Record Forms (CRF). CRFs and other source documents will be kept in locked cabinets. Data will be entered on a regular basis to ensure that it is up to date. The database will be entered on a regular basis on a secure PC, as will the pharmacokinetic database that will be received by the laboratories. Access to the database

will be given to authorized personnel only (members of the immediate study team) and a log of authorized personnel will be stored in the trial master file. CRF and trial documents will be kept in locked cabinets. No participant identifying information will be disclosed in any publication or at any conference activities arising from the study.

12. ETHICS

12.1. Institutional Review Board (IRB)

It is the responsibility of the investigator to have prospective approval of the study protocol, protocol amendments, informed consent documents, and other relevant documents from the IRB. All correspondence with the IRB should be retained in the regulatory or trial master file. Copies of IRB approvals should be filed with other study documents.

12.2. Ethical Conduct of the Study

The study will be conducted in accordance with legal and regulatory requirements, as well as the general principles set forth in the International Ethical Guidelines for Biomedical Research Involving Human Subjects and the Declaration of Helsinki.

In addition, the study will be conducted in accordance with the protocol, GCP guidelines, and applicable local regulatory requirements and laws.

12.3. Subject Information and Consent

All parties will ensure protection of subject personal data and will not include subject names on any forms, reports, publications, or in any other disclosures, except where required by laws.

The informed consent documents used in this study, and any changes made during the course of the study, must be prospectively approved by the IRB.

The investigator, or a person designated by the investigator, will obtain written informed consent from each subject or the subject's legal representative before any study-specific activity is performed. When feasible, the consent process will be conducted in the participants' primary language. If needed, an interpreter will be used to facilitate the process. If the participant is not literate, the consent form will be read aloud to them. Participants will provide informed consent through an electronic signature on a tablet using mobile REDCap if feasible. Alternatively, consent will be obtained on paper. As noted on the consent form, participants may opt to receive a paper copy of their consent form. The investigator will retain the original of each subject's signed consent document.

13. DEFINITION OF END OF TRIAL

The end of the trial is when the last subject visit and last data abstraction is complete.

Post-trial access plan for the study: At the end of the trial, when the last subject visit and last data abstraction is complete, all these participants in the intervention arm will be transitioned to standard of care at respective facilities and those who would wish to continue with community ART delivery models will stay if suitable per current Ministry of health (Uganda) guidelines ~~as is~~ at the end of the trial.

14. PUBLICATION OF STUDY RESULTS

This study will take place over five years. We will start participant recruitment and enrollment at six months and will continue for approximately three years until 3.5 years into the five-year study period. During year two we will work to write and submit to a peer-reviewed journal manuscript #1 describing the Head StART research protocol. In year three, we hope to present an analysis of baseline enrollment data in manuscript #2. In year five we will finalize data collection and analysis. Shortly thereafter, we intend to complete manuscript #3 on Head StART effectiveness for clients newly diagnosed with HIV, manuscript #4 on assessing Head StART using an implementation science framework considering future scaling of the intervention, and manuscript #5 on the cost and budget impact analysis of the Head StART intervention. Throughout the study, semi-annual project updates will be disseminated to stakeholders in Uganda. Presentations will be made at national and international conferences in years 2 through 5. In addition to biannual updates to stakeholders, final results will be disseminated to stakeholders in Uganda and sub-Saharan Africa at the conclusion of the study.

15. CAPACITY BUILDING

The Head StART study will help to build research capacity at the Infectious Diseases Institute (IDI) and within health implementing partner organizations. IDI is a sub-contract recipient of this grant with the Site-PI (Dr. Timothy Muwonge), and Co-Is Drs. Andrew Mujugira and Agnes Kiragga, important members of the core research team. Additionally, over the five-year period of this grant, the project will support an IDI Program Manager, an IDI Project Administrator, an IDI Grants Officer, an IDI Finance Officer and 12 full-time IDI RAs (note: these are the current positions and there may be minor adjustments throughout the grant period as approved by IDI). Further, it is likely that additional people (e.g., graduate students) at IDI will benefit by being involved in this research.

16. FUNDING

National Institute of Mental Health (R01MH130216)

17. REFERENCES

1. O'Laughlin, K.N., et al., *A Social-Ecological Framework to Understand Barriers to HIV Clinic Attendance in Nakivale Refugee Settlement in Uganda: a Qualitative Study.* AIDS and Behavior, 2021. **25**(6): p. 1729-1736.
2. O'Laughlin, K.N., et al., *The cascade of HIV care among refugees and nationals in Nakivale Refugee Settlement in Uganda.* HIV Med, 2017. **18**(7): p. 513-518.
3. Parrish, C., et al., *Social Support and Linkage to HIV Care Following Routine HIV Testing in a Ugandan Refugee Settlement.* AIDS Behav, 2022.
4. Goverment of Uganda Office of the Prime Minister, *Uganda Refugee and Asylum Seekers as of 28 Feb 2022*. 2022.
5. UNAIDS. *Fast track ending the AIDS epdiemic by 2030*. 2014 May 3, 2022]; Available from: https://files.unaids.org/en/media/unaids/contentassets/documents/unaidspublication/2014/20140925_Fast_Track_Brochure.pdf.
6. Decroo, T., et al., *Four-year retention and risk factors for attrition among members of community ART groups in Tete, Mozambique.* Trop Med Int Health, 2014. **19**(5): p. 514-21.
7. Vandendyck, M., et al., *Community-Based ART Resulted in Excellent Retention and Can Leverage Community Empowerment in Rural Lesotho, A Mixed Method Study.* HIV/AIDS Res Treat Open J., 2015. **2**(2): p. 44-50.
8. Bemelmans, M., et al., *Community-supported models of care for people on HIV treatment in sub-Saharan Africa.* Trop Med Int Health, 2014. **19**(8): p. 968-77.
9. Grimsrud, A., et al., *Implementation and Operational Research: Community-Based Adherence Clubs for the Management of Stable Antiretroviral Therapy Patients in Cape Town, South Africa: A Cohort Study.* J Acquir Immune Defic Syndr, 2016. **71**(1): p. e16-23.
10. Pasipamire, L., et al., *Retention on ART and predictors of disengagement from care in several alternative community-centred ART refill models in rural Swaziland.* J Int AIDS Soc, 2018. **21**(9): p. e25183.
11. Kagimu, D., et al. *Overcoming barriers to access of HIV/AIDS services among female sex workers through differentiated service delivery models, TASO Entebbe experience*. in *AIDS 2018*. 2018. Amsterdam, the Netherlands.
12. The Republic of Uganda Ministry of Health. *Implementation Guide for Differentiated Service Delivery Models of HIV Services in Uganda* 2017 May 3, 2022]; Available from: https://differentiatedservicedelivery.org/Portals/0/adam/Content/FXBRYsohBEGGGiGnnnli3A/File/32.%20Differentiated%20Service%20Delivery%20%20Models%20of%20HIV%20Services%20in%20Uganda%20-%20Implementation%20Guide%207.08.2017-1.pdf.
13. Uganda Ministry of Health. *Consolidated Guidelines for the Prevention and Treatment of HIV and AIDS in Uganda*. 2020 May 3, 2022]; Available from: http://library.health.go.ug/publications/hivaids/consolidated-guidelines-prevention-and-treatment-hiv-uganda.
14. World Health Organization (WHO). *HIV/AIDS*. May 3, 2022]; Available from: https://www.afro.who.int/health-topics/hivaids.
15. Pew Research Center. *Record number of forcibly displaced people lived in sub-Saharan Africa in 2017*. 2018 May 3, 2022]; Available from: https://www.pewresearch.org/fact-tank/2018/08/09/record-number-of-forcibly-displaced-people-lived-in-sub-saharan-africa-in-2017/.
16. United Nations High Commissioner for Refugees (UNHCR). *Africa*. May 3, 2022]; Available from: https://www.unhcr.org/en-us/africa.html.
17. UNAIDS and UNHCR. *Strategies to support the HIV related needs of refugees and host populations*. 2005 May 3, 2022]; Available from: http://data.unaids.org/publications/irc-pub06/jc1157-refugees_en.pdf.

18. O'Laughlin, K.N., et al., *Testing experiences of HIV positive refugees in Nakivale Refugee Settlement in Uganda: informing interventions to encourage priority shifting.* Confl Health, 2013. **7**(1): p. 2.
19. Tanaka, Y., et al., *Knowledge, attitude, and practice (KAP) of HIV prevention and HIV infection risks among Congolese refugees in Tanzania.* Health Place, 2008. **14**(3): p. 434-52.
20. United Nations High Commissioner for Refugees (UNHCR). *Operational guidance mental health & psychosocial support programming for refugee operations*. 2013 May 3, 2022]; Available from: http://www.unhcr.org/525f94479.pdf.
21. O'Laughlin, K.N., et al., *A qualitative approach to understand antiretroviral therapy (ART) adherence for refugees living in Nakivale Refugee Settlement in Uganda.* Confl Health, 2018. **12**: p. 7.
22. UNAIDS and UNHCR. *Policy Brief: HIV and refugees*. 2007 May 3, 2022]; Available from: http://data.unaids.org/pub/briefingnote/2007/policy_brief_refugees.pdf.
23. UNAIDS. *Understanding fast-track: Accelerating action to end the AIDS epidemic by 2030*. May 3, 2022]; Available from: https://www.unaids.org/sites/default/files/media_asset/201506_JC2743_Understanding_FastTrack_en.pdf.
24. United Nations High Commissioner for Refugees. *Refugee Data Finder*. 2022 May 3, 2022]; Available from: https://www.unhcr.org/refugee-statistics/.
25. International AIDS Society (IAS). *Differentiated Service Delivery*. May 3, 2022]; Available from: http://www.differentiatedservicedelivery.org/about.
26. Auld, A.F., et al., *A Decade of Antiretroviral Therapy Scale-up in Mozambique: Evaluation of Outcome Trends and New Models of Service Delivery Among More Than 300,000 Patients Enrolled During 2004-2013.* J Acquir Immune Defic Syndr, 2016. **73**(2): p. e11-22.
27. Kun, K.E., et al., *Mozambique's Community Antiretroviral Therapy Support Group Program: The Role of Social Relationships in Facilitating HIV/AIDS Treatment Retention.* AIDS Behav, 2019. **23**(9): p. 2477-2485.
28. Fatti, G., et al., *Outcomes of Three- Versus Six-Monthly Dispensing of Antiretroviral Treatment (ART) for Stable HIV Patients in Community ART Refill Groups: A Cluster-Randomized Trial in Zimbabwe.* J Acquir Immune Defic Syndr, 2020. **84**(2): p. 162-172.
29. Barnabas, R.V., et al., *Community-based antiretroviral therapy versus standard clinic-based services for HIV in South Africa and Uganda (DO ART): a randomised trial.* Lancet Glob Health, 2020. **8**(10): p. e1305-e1315.
30. Sharp, J., et al., *Outcomes of patients enrolled in an antiretroviral adherence club with recent viral suppression after experiencing elevated viral loads.* South Afr J HIV Med, 2019. **20**(1): p. 905.
31. Okiror, A. *ART adherence among patients cared in the community client-led ART delivery (CCLAD) - TASO, Uganda experience*. in *AIDS 2018*. 2018. Amsterdam, the Netherlands.
32. Sallis, J., N. Owen, and E. Fisher, *Ecological models of health behavior*, in *Health Behavior and Health Education: Theory, Research, and Practice*, K. Glanz, B. Rimer, and F. Lewis, Editors. 2008, John Wiley & Sons: San Francisco, California, USA. p. 465-486.
33. Stokols, D., *Translating social ecological theory into guidelines for community health promotion.* Am J Health Promot, 1996. **10**(4): p. 282-98.
34. Stokols, D., *Establishing and maintaining healthy environments. Toward a social ecology of health promotion.* Am Psychol, 1992. **47**(1): p. 6-22.
35. Bronfenbrenner, U., *Ecological systems theory*. 1992, Philadelphia, PA: Jessica Kingsley Publishers.
36. McLeroy, K.R., et al., *An ecological perspective on health promotion programs.* Health Educ Q, 1988. **15**(4): p. 351-77.
37. Galea, J.T., et al., *Barriers and facilitators to antiretroviral therapy adherence among Peruvian adolescents living with HIV: A qualitative study.* PLoS One, 2018. **13**(2): p. e0192791.
38. Saleem, H.T., et al., *Achieving pregnancy safely for HIV-serodiscordant couples: a social ecological approach.* J Int AIDS Soc, 2017. **20**(Suppl 1): p. 21331.

39. Glick, J.L., et al., *ART uptake and adherence among female sex workers (FSW) globally: A scoping review.* Glob Public Health, 2020: p. 1-31.
40. Christian, S.N., et al., *Applying a social-ecological lens to opinions about HIV self-testing among Kenyan truckers who declined to test: a qualitative study.* Afr J AIDS Res, 2020. **19**(2): p. 147-155.
41. Scheibe, A., et al., *Money, power and HIV: economic influences and HIV among men who have sex with men in sub-Saharan Africa.* Afr J Reprod Health, 2014. **18**(3 Spec No): p. 84-92.
42. Katz, I.T., et al., *Treatment guidelines and early loss from care for people living with HIV in Cape Town, South Africa: A retrospective cohort study.* PLoS Med, 2017. **14**(11): p. e1002434.
43. Puttkammer, N., et al., *ART attrition and risk factors among Option B+ patients in Haiti: A retrospective cohort study.* PLoS One, 2017. **12**(3): p. e0173123.
44. Bassett, I.V., et al., *Routine, voluntary HIV testing in Durban, South Africa: correlates of HIV infection.* HIV Med, 2008. **9**(10): p. 863-7.
45. Lewis, M.A., et al., *Using RE-AIM to examine the potential public health impact of an integrated collaborative care intervention for weight and depression management in primary care: Results from the RAINBOW trial.* PLoS One, 2021. **16**(3): p. e0248339.
46. Iwelunmor, J., et al., *Is it time to RE-AIM? A systematic review of economic empowerment as HIV prevention intervention for adolescent girls and young women in sub-Saharan Africa using the RE-AIM framework.* Implement Sci Commun, 2020. **1**: p. 53.
47. Brant, A.R., et al., *Integrating HIV Pre-Exposure Prophylaxis into Family Planning Care: A RE-AIM Framework Evaluation.* AIDS Patient Care STDS, 2020. **34**(6): p. 259-266.
48. Carroll, C., et al., *A conceptual framework for implementation fidelity.* Implement Sci, 2007. **2**: p. 40.
49. Songtaweesin, W.N., et al., *Youth-friendly services and a mobile phone application to promote adherence to pre-exposure prophylaxis among adolescent men who have sex with men and transgender women at-risk for HIV in Thailand: a randomized control trial.* J Int AIDS Soc, 2020. **23 Suppl 5**: p. e25564.
50. Cover, J., et al. *Individual and contextual factors associated with PrEP adherence among Kenyan adolescent girls and young women*. in *International AIDS Society*. 2020.
51. National Institute of Allergy and Infectious Diseases (NIAID). *Division of AIDS (DAIDS) Table for Grading the Severity of Adult and Pediatric Adverse Events*. 2017 August 17, 2022]; Available from: https://rsc.niaid.nih.gov/clinical-research-sites/daids-adverse-event-grading-tables.
52. Mendelsohn, J.B., et al., *Low levels of viral suppression among refugees and host nationals accessing antiretroviral therapy in a Kenyan refugee camp.* Confl Health, 2017. **11**: p. 11.
53. Magidson, J.F., et al., *Project Khanya: a randomized, hybrid effectiveness-implementation trial of a peer-delivered behavioral intervention for ART adherence and substance use in Cape Town, South Africa.* Implement Sci Commun, 2020. **1**.
54. Barnabas, R.V., et al., *Initiation of antiretroviral therapy and viral suppression after home HIV testing and counselling in KwaZulu-Natal, South Africa, and Mbarara district, Uganda: a prospective, observational intervention study.* Lancet HIV, 2014. **1**(2): p. e68-e76.
55. Uganda Ministry of Health and UPHIA 2016-2017 Collaborating Institutions. *Uganda Population-Based HIV Impact Assessment* 2019 May 3, 2022]; Available from: https://phia.icap.columbia.edu/wp-content/uploads/2019/07/UPHIA_Final_Report_Revise_07.11.2019_Final_for-web.pdf.
56. Hayes, R.J., et al., *Effect of Universal Testing and Treatment on HIV Incidence - HPTN 071 (PopART).* N Engl J Med, 2019. **381**(3): p. 207-218.
57. Sikazwe, I., et al., *Retention and viral suppression in a cohort of HIV patients on antiretroviral therapy in Zambia: Regionally representative estimates using a multistage-sampling-based approach.* PLoS Med, 2019. **16**(5): p. e1002811.
58. Cohen, M.S., et al., *Prevention of HIV-1 infection with early antiretroviral therapy.* N Engl J Med, 2011. **365**(6): p. 493-505.

59. Cohen, M.S., et al., *Antiretroviral Therapy for the Prevention of HIV-1 Transmission.* N Engl J Med, 2016. **375**(9): p. 830-9.
60. Rodger, A.J., et al., *Sexual Activity Without Condoms and Risk of HIV Transmission in Serodifferent Couples When the HIV-Positive Partner Is Using Suppressive Antiretroviral Therapy.* JAMA, 2016. **316**(2): p. 171-81.
61. Bavinton, B.R., et al., *Viral suppression and HIV transmission in serodiscordant male couples: an international, prospective, observational, cohort study.* Lancet HIV, 2018. **5**(8): p. e438-e447.
62. Hsieh, H.F. and S.E. Shannon, *Three approaches to qualitative content analysis.* Qual Health Res, 2005. **15**(9): p. 1277-88.
63. Palinkas, L.A., et al., *Mixed method designs in implementation research.* Adm Policy Ment Health, 2011. **38**(1): p. 44-53.
64. Ivankova, N.V., J. Creswell, and S.L. Stick, *Using mixed-methods sequential explanatory design: from theory to practice.* Field Methods, 2006. **18**(1): p. 3-20.
65. Fetters, M.D., L.A. Curry, and J.W. Creswell, *Achieving integration in mixed methods designs-principles and practices.* Health Serv Res, 2013. **48**(6 Pt 2): p. 2134-56.
66. Morse, J.M., *Approaches to qualitative-quantitative methodological triangulation.* Nurs Res, 1991. **40**(2): p. 120-3.
67. Sullivan, S.D., et al., *Budget impact analysis-principles of good practice: report of the ISPOR 2012 Budget Impact Analysis Good Practice II Task Force.* Value Health, 2014. **17**(1): p. 5-14.
