# Supplementary material for: Protocol for Head StART: A hybrid type II cluster randomized controlled trial evaluating community ART delivery for people newly diagnosed with HIV in refugee settlements in Uganda
Source: PLoS One. 2026 Feb 27;21(2):e0340916. doi: 10.1371/journal.pone.0340916 (PMC12948099; doi:10.1371/journal.pone.0340916)
Supplement: S4 File — (DOCX) [file pone.0340916.s004.docx]

Inclusivity in global research

PLOS’ policy on inclusivity in global research aims to improve transparency in the reporting of research performed outside of researchers’ own country or community and ensures that PLOS publications reporting global research adhere to high standards for research ethics and authorship. Authors of relevant research articles may be asked to complete the questionnaire below, which outlines ethical, cultural, and scientific considerations specific to inclusivity in global research. This questionnaire may be requested when researchers have travelled to a different country to conduct research, if research uses samples collected in another country, research with Indigenous populations or their lands, or if research is on cultural artefacts. Researchers travelling to another country solely to use laboratory equipment will not normally be required to complete the questionnaire. However, the questionnaire can be requested at the journal’s discretion for any submission – if you have been requested to complete this questionnaire by the PLOS journal you submitted to, please do so.

Please complete the questionnaire below and include this as a Supporting Information file with your manuscript. Note that if your paper is accepted for publication, this checklist will be published with your article in the supporting information files. Please ensure that you reference the checklist in the main body of your manuscript. We suggest adding a subsection ‘Inclusivity in global research’ to your Methods section and adding the following sentence: “Additional information regarding the ethical, cultural, and scientific considerations specific to inclusivity in global research is included in the Supporting Information (SX Checklist)”

The questions have been designed to be applicable to a wide range of study types, and there are subsections for both human subjects research and non-human subjects research. If any of the questions are not relevant to your research please mark them as “N/A” as appropriate.

**Ethical considerations, permits and authorship**

*This section is applicable to all research types.*

Provide details as to who granted permissions and/or consent for the study to take place in the Methods section of your manuscript. This should include the names of **all** ethics boards, governmental organizations, community leaders or other bodies that provided approval for the study. If individuals provided approval refer to these people by their role or title but do not list their name(s).

This study was approved by the Makerere University Infectious Diseases Institute Research Ethics Committee and the University of Washington Human Subjects Institutional Review Board (manuscript, pages 20 - 21). Additional approval was obtained from the Uganda National Council for Science and Technology. Support for the study was granted from the Refugee Desk Officer in the Uganda Office of the Prime Minister and the AIDS Control Program Manager in the Uganda Ministry of Health.

If there were any deviations from the study protocol after approval was obtained please provide details of these changes in the Methods section of your manuscript.
Did this study involve local collaborators that are residents of the country where the research was conducted or members of the community studied? If you do not have any authors from said communities, please provide an explanation for this below.

Yes – authors Ambrose Mugyenyi, Rogers Nsubuga, Glori Asaba, Zikama Faustin, Andrew Mujugira, and Timothy Muwonge are residents of Uganda.

**Protocol revisions were made following initial approval, and all revisions were reviewed and approved by both the Makerere University Infectious Diseases Institute Research Ethics Committee and the University of Washington Human Subjects Institutional Review Board. In addition to several minor edits to improve clarity of language and study procedures, substantive revisions included the following:**

1. **Randomization:** Changed from matched-pair to stratified-pair randomization to optimize statistical efficiency.
2. **Sample size:** Updated sample size calculations to reflect the revised randomization approach and to account for expected loss to follow-up.
3. **Eligibility criteria:** Revised inclusion and exclusion criteria to promote inclusivity and improve clarity. Inclusion criteria were expanded to include mature and emancipated minors. Exclusion criteria were narrowed from excluding all participants with comorbidities or higher WHO clinical stages to excluding only those whom clinicians deemed to require facility-based care. We also clarified that clients testing HIV-positive in the prior six months were eligible only if they were not previously known to be HIV-positive.
4. **Data monitoring committee:** Revised language regarding DMC composition.
5. **Laboratory procedures:** Clarified tenofovir testing language and procedures for dried blood spot collection, testing, and storage.
6. **Data collection:** Updated plans for direct observation data collection.
7. **Reporting:** Revised reporting requirements based on feedback from the DMC and IRB.
8. **Analysis plan:** Extended the allowable window for viral load and tenofovir level outcomes from ±2 months to ±3 months.

These deviations are described in the Methods section on pages 21 - 22.

Everyone listed as an author should meet PLOS’ criteria for authorship and all individuals who meet these criteria should be included in the author byline, rather than the acknowledgements. For further information please see the journal’s Authorship Policy.

**Human subjects research (e.g. health research, medical research, cross-cultural psychology)**

Did you obtain written informed consent from a representative of the local community or region before the research took place? How did you establish who speaks for the community? Details of written informed consent obtained from study participants should be reported separately in the Methods section of your manuscript.

We sought guidance during the study design and implementation phase from community members, local academicians and local stakeholeders (leaders at the Ministry of Health and in the United Nations High Commissioner for Refugees Uganda). Additionally, we obtained permission to conduct this work from the Makerere University Infectious Diseases Institute Research Ethics Committee, the Uganda National Council for Science and Technology. Before we began we had support for the study from the Refugee Desk Officer in the Uganda Office of the Prime Minister and the AIDS Control Program Manager in the Uganda Ministry of Health. We did not obtain written informed consent from a representative of the local community or region before the research took place.

How did members of the local community provide input on the aims of the research investigation, its methodology, and its anticipated outcome(s)?

Health workers in the study setting and key local stakeholders—including representatives from the Ministry of Health, the United Nations High Commissioner for Refugees Uganda, and Medical Teams International—were invited to the study inception meeting and early stakeholder discussions to provide feedback on the study design. Their input led to revisions of the study inclusion and exclusion criteria. Specifically, the age range was expanded to include people living with HIV who were younger than 18 years but considered mature or emancipated minors, and exclusion criteria regarding comorbidities and higher WHO clinical stages were narrowed to only those whom clinicians deemed to require facility-based care. It was clarified that clients testing HIV-positive in the prior six months were eligible only if they were not previously known to be HIV-positive.

Input from community members during Community Advisory Board meetings informed the qualitative interview guides by highlighting additional areas for deeper exploration. These discussions also helped catalyze the effort to document ongoing adaptations to community ART delivery throughout the study.

When engaging with the local community, how did you ensure that the informed consent documents and other materials could be understood by local stakeholders?

Drafts of data collection tools, including qualitative interview guides, were reviewed by research assistants working in the study setting, and their feedback informed revisions to improve clarity, cultural relevance, and appropriateness. All study tools were translated into Kiswahili, Kinyarwanda, Runyankore, and Somali—languages commonly spoken in the setting. When engaging with participants in languages in which research assistants are not fluent, trained interpreters working in the study setting provide support.

Will the findings of the research be made available in an understandable format to stakeholders in the community where the study was conducted (e.g. via a presentation, summary report, copies of publications, etc.)? Please provide details of how this will be achieved.

Dissemination of study progress and findings will take place throughout the course of the study, as well as at conclusion of the study. Over the course of study implementation, stakeholders in Uganda receive updates on study progress through virtual monthly study meetings as well as community advisory board (CAB) meetings occurring 2-4 times a year (see manscript page 10 for further description of CABs). Final results of the study will also be disseminated to local stakeholders.

**Non-human subjects research using specimens/ animals collected as part of the study, or those housed in archival collections. Examples include archaeology, paleontology, botany and zoology.**

Did the permission you obtained from a local authority to perform the study include an agreement on access to outputs and benefit sharing? This may include procedures to enable fair distribution of the benefits and resources arising from the research performed. Please include any details of Prior Informed Consent and Benefit Sharing Agreements obtained. These may be required by field-specific regulations, for example the Convention on Biological Diversity (CBD) and the associated Nagoya Protocol.

N/A

If the material used in your study was imported, please A) provide the year it was imported and B) indicate whether permits were obtained to import/export the materials used, C) provide details of any permits obtained. If this information is not available, please indicate this.

N/A

If you used archival specimens, please state how the material used in your study was acquired by the institute it is held in and provide details of any permits obtained for the original excavations/ sample collection. If this information is not available, please indicate this.

N/A

How was the potential cultural significance of the materials collected in your study to local communities considered in your research design? Were Indigenous peoples and/or local researchers and institutions involved with archaeological excavations / collection of specimens? If so, please provide a description of their involvement.

N/A

If your manuscript includes photographs of human remains please indicate whether authors obtained permission from descendants or affiliated cultural communities to do so.

N/A
